# Focused electron beam induced deposition: A perspective

**DOI:** 10.3762/bjnano.3.70

**Published:** 2012-08-29

**Authors:** Michael Huth, Fabrizio Porrati, Christian Schwalb, Marcel Winhold, Roland Sachser, Maja Dukic, Jonathan Adams, Georg Fantner

**Affiliations:** 1 Physikalisches Institut, Max-von-Laue-Str. 1, Goethe-Universität, 60438 Frankfurt am Main, Germany; 2 Institute of Bioengineering, EPFL, STI IBI-STI LBNI, BM 3109 (Bâtiment BM), Station 17, CH-1015 Lausanne, Switzerland

**Keywords:** atomic force microscopy, binary systems, electron beam induced deposition, granular metals, micro Hall magnetometry, radiation-induced nanostructures, strain sensing

## Abstract

**Background:** Focused electron beam induced deposition (FEBID) is a direct-writing technique with nanometer resolution, which has received strongly increasing attention within the last decade. In FEBID a precursor previously adsorbed on a substrate surface is dissociated in the focus of an electron beam. After 20 years of continuous development FEBID has reached a stage at which this technique is now particularly attractive for several areas in both, basic and applied research. The present topical review addresses selected examples that highlight this development in the areas of charge-transport regimes in nanogranular metals close to an insulator-to-metal transition, the use of these materials for strain- and magnetic-field sensing, and the prospect of extending FEBID to multicomponent systems, such as binary alloys and intermetallic compounds with cooperative ground states.

**Results:** After a brief introduction to the technique, recent work concerning FEBID of Pt–Si alloys and (hard-magnetic) Co–Pt intermetallic compounds on the nanometer scale is reviewed. The growth process in the presence of two precursors, whose flux is independently controlled, is analyzed within a continuum model of FEBID that employs rate equations. Predictions are made for the tunability of the composition of the Co–Pt system by simply changing the dwell time of the electron beam during the writing process. The charge-transport regimes of nanogranular metals are reviewed next with a focus on recent theoretical advancements in the field. As a case study the transport properties of Pt–C nanogranular FEBID structures are discussed. It is shown that by means of a post-growth electron-irradiation treatment the electronic intergrain-coupling strength can be continuously tuned over a wide range. This provides unique access to the transport properties of this material close to the insulator-to-metal transition. In the last part of the review, recent developments in mechanical strain-sensing and the detection of small, inhomogeneous magnetic fields by employing nanogranular FEBID structures are highlighted.

**Conclusion:** FEBID has now reached a state of maturity that allows a shift of the focus towards the development of new application fields, be it in basic research or applied. This is shown for selected examples in the present review. At the same time, when seen from a broader perspective, FEBID still has to live up to the original idea of providing a tool for electron-controlled chemistry on the nanometer scale. This has to be understood in the sense that, by providing a suitable environment during the FEBID process, the outcome of the electron-induced reactions can be steered in a controlled way towards yielding the desired composition of the products. The development of a FEBID-specialized surface chemistry is mostly still in its infancy. Next to application development, it is this aspect that will likely be a guiding light for the future development of the field of focused electron beam induced deposition.

## Review

### Introduction

Focused electron beam induced deposition (FEBID) is receiving strongly increasing attention as a direct-writing technique for nanostructures due to its great versatility. In FEBID a previously adsorbed molecular precursor is dissociated in the focus of an electron beam provided by a scanning or transmission electron microscope (SEM/TEM). By and large, the focal area diameter of the electron beam, convoluted by the surface-leaving secondary electrons, determines the lateral resolution of this method. Resolutions better than 3 nm in SEMs [1] and even below 1 nm in TEMs [2] have been shown to be feasible. Due to this excellent resolution, FEBID, with the extension of focused electron beam induced etching (FEBIE), is now the de facto standard in mask repair for the 193 nm node [3]. It also holds great promise for circuit editing. Several reviews have been published in recent years [4–5] discussing various aspects of FEBID, or focused electron beam induced processing (FEBIP), the most comprehensive of which is the excellent article by Utke, Hoffmann and Melngailis [6]. These reviews mainly cover the principles of gas-assisted deposition and etching with electrons, provide a summary of modeling approaches to FEBIP, and give some details of the various characterization techniques for FEBID structures. Application fields in research are discussed with a strong view to potential uses in industry.

In this review, some very recent developments in FEBID-based research are presented. In this context we limit the presentation to an interrelated group of topics covering the importance of granular metals obtained from FEBID for basic research in correlation physics, as well as the potential for application of these granular metals in magnetic and strain sensing. Furthermore, the extensibility of FEBID to the preparation of binary metals is discussed with a prospect of directly writing a wider range of magnetic or superconducting structures on the nanometer scale. After a very brief discourse of the FEBID process presented in the next section, the modeling of FEBID on the basis of rate equations is discussed with a view to more than one precursor being present during the process. This leads on to the third section, which presents some recent results on the preparation and characterization of binary FEBID structures, with special focus on magnetism and superconductivity. The following section reviews the particular advantages that FEBID structures provide in resolving long-standing issues in the physics of nanogranular metals close to the metal–insulator transition. The implications of the nanogranular microstructure, often obtained in FEBID, for sensor applications are subsequently presented. Nanogranular structures, i.e., structures that contain metal nanocrystallites embedded in a dielectric matrix, have special properties that make them particularly suitable for magnetic-field- and strain-sensing applications. The conclusion will present our views on the challenges that FEBID will have to face in the near to midterm future.

### FEBID: Brief review of the fundamentals

**FEBID in a nutshell**: The FEBID process is based on the electron-induced dissociation of a molecular precursor previously adsorbed on a substrate surface and constantly replenished by a gas-supply system. In most instances the gas-supply or gas-injection system consists of a precursor reservoir that can be heated or cooled, and which is coupled to a fine capillary with a typical diameter of 0.5 mm. The open end of the capillary can be brought into close proximity to the substrate surface on which the electron beam is focused.

**Figure 1:**
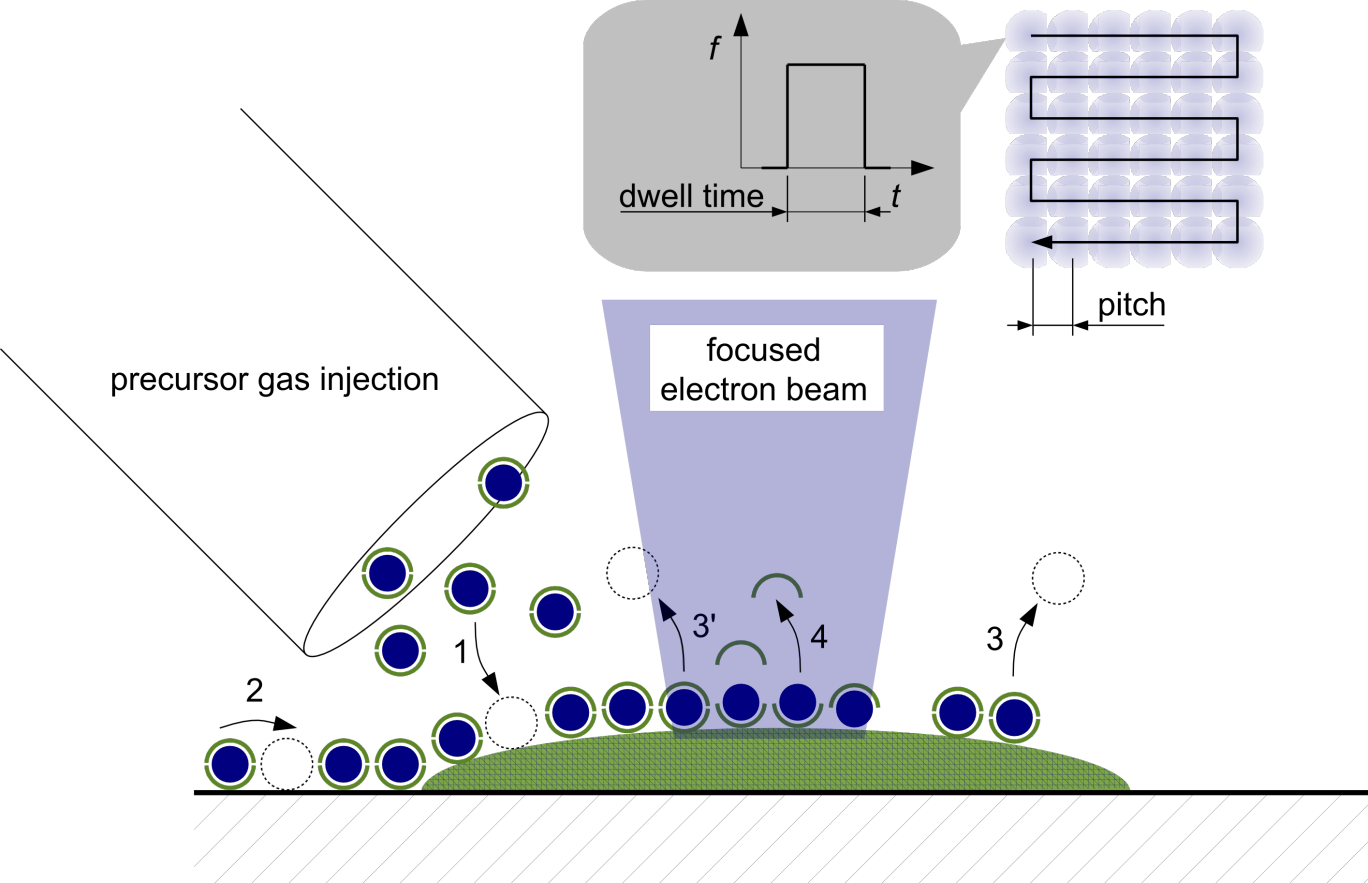
Illustration of FEBID. Precursor molecules (here: organometallic complex; blue: metal, green: organic ligands) are supplied by a gas-injection system and physisorb (1) on the surface. Surface diffusion (2), thermally induced desorption (3) and electron-stimulated desorption (3') take place. Within the focus of the electron beam, adsorbed precursor molecules are (partly) dissociated followed by desorption of volatile organic ligands (4). Upper right: For pattern definition the electron beam is moved in a raster fashion (here: serpentine) over the surface and settles on each dwell point for a specified dwell time. After one raster sequence is completed the process is repeated until a predefined number of repeated loops is reached.

**Technical parameters**: The main parameters that govern the writing process are the primary-beam energy *E* and beam current *I*, the time for which the electron beam is held constant on a particular point on the surface, the dwell time *t_D_*, the distance between neighboring dwell points, the pitch *p*, and the number of loops for which the writing pattern is repeated, *n_L_*. Further important parameters are the replenishment time, *t_r_*, i.e., the time period for which the writing is paused between two successive loops, and the geometry of the writing path, i.e., zig-zag, meander or spiral, to list the most commonly used. Figure 1 gives a graphical overview of the FEBID process.

**Precursor chemistry**: Suitable precursors for the FEBID process, which mostly takes place with the precursor and substrate temperature close to room temperature, need to have sufficiently high vapor pressures in the temperature range of about 270 K to 320 K. A typical vapor pressure would be 10^−2^ to 10 mbar for organometallic precursors, but this can only serve as a very crude guideline. A very detailed account on FEBID precursors and their properties can be found in Utke et al. [6], ordered according to the respective type of organic ligand. Quite generally speaking, once supplied to the substrate a precursor must have a sufficiently long residence time τ on the surface, typically lasting microseconds to milliseconds. Otherwise, at typical energy-dependent dissociation cross sections σ(*E*) of 10^−3^ to 10^−2^ nm^2^ in the energy range below 500 eV for metal–organic precursors, the deposition yield would be too small. On the other hand, the volatile organic dissociation products should readily desorb to prevent their usually undesired inclusion in the deposit. Depending on the targeted functionality of the FEBID structure, several different precursor classes are employed, such as alkanes, silanes, metal halogens, carbonyls, phosphines, acetylacetonates and so forth. In the following the focus is on organometallic precursors. Popular representatives for the transition metals are carbonyls, such as W(CO)_6_ or Co_2_(CO)_8_, but also more complex precursors, such as Me_3_Pt(IV)CpMe. For details the reader is referred to Utke et al. [6]. With a view to the following sections silane-based precursors, such as neo-pentasilane Si_5_H_12_, are also of interest. The chemical-bond structure is highly relevant for the details of the electron-induced dissociation process, which is discussed next.

**Electron-induced dissociation**: Many different electron–molecule interaction processes are relevant for FEBID. They can be summarized as shown in Table 1.

**Table 1:** Electron–molecule interaction processes relevant for FEBID according to [3]. Rotational excitations are not explicitly included. *E_i_*: initial kinetic energy of electron, *E_r_*: residual kinetic energy of electron after process.

	process type

*e*^−^(*E_i_*) + AB → AB + *e*^−^(*E_i_*)	elastic scattering
*e*^−^(*E_i_*) + AB → AB(*ν*) + *e*^−^(*E_r_*)	vibrational excitation (VE)
*e*^−^(*E_i_*) + AB → AB^*^ + *e*^−^(*E_r_*)	electronic excitation (EE)
*e*^−^ + AB → A^•^ + B^−^	dissociative electron attachment (DEA)
*e*^−^ + AB → A^•^ + B^•^ + *e*^−^	neutral dissociation (ND)
*e*^−^ + AB → A^•^ + B^+^ + 2*e*^−^	dissociative ionization (DI)
*e*^−^ + AB → A^−^ + B^+^ + *e*^−^	bipolar dissociation / ion pair formation (BD)

Depending on the energy range, various different specialized instruments have to be applied to acquire absolute energy-dependent cross sections for these processes. For FEBID the relevant energy range is 1 meV (slowed-down secondary electrons) up to the keV regime (typical primary electron regime, forward and backscattered electrons). As a consequence, complete cross section data sets are very rare, and this is in particular the case for precursors commonly used in FEBID. In particular, one has to keep in mind that dissociation cross sections obtained on precursor molecules in the gas phase do not necessarily provide a suitable basis for a quantitative description of precursors in an adsorbed state. The coupling to the substrate provides additional relaxation channels for both electronic and vibronic excitations. As an additional complexity, one has to note that several relevant cross sections, such as those of transient molecules produced in FEBID, are very hard to measure or may even be inaccessible to quantification. Therefore, theoretical advancements in calculating reliable energy-dependent cross sections are of special importance. At the present stage it is fair to say that for none of the precursors commonly used in FEBID is a full set of data of energy-dependent cross sections available, although some energy-dependent data for a few precursors can be found in the literature [3,6]. The reader is referred to Utke et al. [3] for a detailed account on the fundamentals of the interactions of electrons with molecules relevant for FEBID.

As a consequence of the lack of reliable energy-dependent cross-section data, in all attempts at modeling FEBID effectively, energy-integrated dissociation cross sections are used. These can be self-consistently obtained from the modeling approach, by comparison with the experimentally determined deposition yields. An additional important aspect is that previously deposited material is constantly irradiated as the deposition progresses since the electrons typically penetrate at least 100 nm into the grown structures at the often-employed primary energy of 5 keV. Nondissociated precursor fragments, which have been embedded in the deposit during the FEBID process, can thus become subject to post-growth dissociation. As a matter of fact, post-growth irradiation can be advantageously used for fine-tuning the electronic transport properties of FEBID structures, and this will be discussed in the context of nanogranular structures later in this review. In any case, the energy spectrum of the electrons that can take part in the FEBID process is important and will be briefly reviewed in the following paragraph.

**Spectrum of relevant electrons**: Assuming an aberration-free primary electron beam with proper astigmatic correction, the radial flux distribution impinging on a plane surface has the shape of a Gaussian

[1]
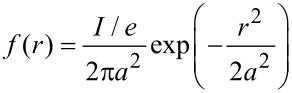


where *f*(*r*) defines the radial flux per unit time and area, and *a* is the standard deviation. As a possible measure of the focal diameter of the beam, the full width at half maximum (FWHM) can be used, which amounts to 
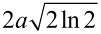
 ≈ 2.36*a*. These primary electrons are subject to interactions with the precursor molecules but also with the underlying substrate. They generate secondary electrons, which are produced by inelastic collisions with the weakly bound valence electrons in the substrate or previously grown deposit. In general, the spectrum of secondary electrons depends on the properties of the substrate material and is characterized by a maximum in the low-energy region from 1 to 10 eV, followed by a tail to higher energies that roughly decreases with the third power of *E*.

An idealized spectral shape was suggested by Chung and Everhart [7], which does in fact provide a reasonable description of the higher-energy tail:

[2]
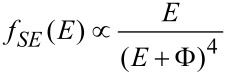


with the work function Φ. For FEBID, in particular with a view to the microstructure of the typically obtained inhomogeneous deposits, which then act as a “growing substrate”, the spectrum of secondary electrons is a priori unknown. Considering the fact that the radial density distribution of the surface-leaving electrons is very important for FEBID, in particular since the dissociation cross sections tend to be larger at low energies, Monte Carlo simulations, describing in detail the electron transport in the substrate and deposit, provide important insights [8–9]. For the purpose of the present review it suffices to state that it is mainly the lateral range of the surface-leaving secondary electrons that limits the resolution of FEBID. Nevertheless, sub-3 nm resolution is achievable in high-resolution SEMs for small-aspect-ratio structures [2]. The electron flux used in the FEBID modeling approach presented in the next section effectively incorporates the contribution of secondary electrons. The influence of forward-scattered electrons is important for high-aspect-ratio FEBID structures and can be properly accounted for in Monte Carlo simulations [8–9].

**Deposit microstructure**: The microstructure of materials obtained by FEBID falls into the three categories amorphous, nanogranular or nanocomposite and polycrystalline. Depending on the microstructure the physical properties vary substantially, e.g., with regard to electrical transport, magnetism or the mechanical strength. Since the local growth rates can be very high, reaching tens to hundreds of nanometers per second, growth proceeds far from equilibrium and is mainly kinetically controlled. A qualitative understanding of the processes resulting in these microstructure classes can be gained from modeling the evolution of phase boundaries in solids at the nanoscale. The formation of a nanogranular microstructure in particular can be understood in the framework of Cahn–Hilliard-like equations applied to such aspects as spinodal decomposition or nucleation [10]. At this point a similarity to the microstructure formation processes in the growth of diamond-like carbon (DLC) films with metal additives can be stated [11]. A distinct difference between FEBID and DLC thin-film research is of course that, in most instances, attempts are made to tune the FEBID process such that carbon inclusion in the deposit can be avoided, whereas in DLC thin-films the carbon component is essential with regard to the desired mechanical or electrical properties. Nevertheless, considering the substantial amount of literature devoted to DLC research, much can be learned concerning the microstructure formation processes. In FEBID it can be observed that organometallic precursors with metal atoms having a tendency to carbide formation result mainly in amorphous deposits, whereas precursors with metals that are immiscible with carbon tend to yield nanogranular structures, i.e., they form metallic nanocrystallites embedded in an amorphous, carbonaceous matrix. In DLC thin-film growth, which is mostly done by reactive sputtering in a mixed Ar and acetylene gas atmosphere from a metallic target, analogous observations are made with regard to the microstructure depending on the miscibility of the target metal with carbon. The granular microstructure is most interesting for basic research on nanogranular metals as artificial nanosolids, in particular if the electronic coupling strength between the metallic grains can be tuned through the insulator-to-metal transition. The exact nature of this transition in three spatial dimensions is not known yet [12]. Also with regard to sensor applications nanogranular materials prepared by FEBID hold great promise. These aspects will be discussed in later chapters of this review. For selected precursors, such as Co_2_(CO)_8_ [13], Fe(CO)_5_ [14–15] and also AuClPF_3_ [16], polycrystalline deposits can be obtained with only small carbon impurity contributions.

So far the complexity of the beam-induced chemical reaction pathways is too large to allow us to develop a detailed understanding of the microscopic formation processes that result in a particular microstructure and elemental composition. Very few surface-science-oriented experiments that try to get an understanding of the deposition process on the molecular level have been performed under well-controlled conditions, such as ultrahigh vacuum. A recent brief overview of this research can be found in Wnuk et al. [17]. Also, initial steps in the analysis, by theoretical means, of the adsorption process of commonly used precursors on thermally grown SiO_2_ surfaces, often employed in FEBID, have only recently been taken within a density functional approach including van der Waals corrections [18–20].

### FEBID modeling

To date no attempts have been made to realistically simulate the nanostructure formation process during FEBID. This must remain a goal for the future. What has been achieved is the modeling of process rates and the simulation of growth geometries. Process rate calculations are almost solely based on continuum models that rely on differential equations for the rate of change of adsorbates relevant for the FEBID process. This will be the focus of this section, with particular emphasis on employing this approach to multicomponent scenarios relevant for the formation of binary FEBID structures, i.e., structures grown in the presence of two different precursor species. The modeling of growth geometries is mainly done by Monte Carlo approaches and allows for integrating the simulation of the electron–solid interaction processes with the surface-based dissociation rates at the cost of a substantially larger numerical complexity [21].

#### Single precursor species continuum model of FEBID

The single precursor species continuum model of FEBID assumes a weak precursor–substrate interaction of the van der Waals type and relies on a Langmuir adsorption description neglecting possible interactions between the adsorbed precursor molecules. The surface coverage is assumed to be limited to one monolayer, such that the maximum fractional coverage *n*/*n_ML_* is 1, where *n_ML_* stands for the full area density of a complete precursor monolayer and *n* for the temporally and spatially dependent precursor adsorbate density. The fraction of surface sites that is available for adsorption is therefore 1 − *n*/*n_ML_*. The model also includes surface diffusion, with diffusion constant *D*, and an average residence time τ for the precursor molecules before desorption. It furthermore takes into account the electron-induced dissociation leading to a reduction of the adsorbate density assuming an energy-integrated dissociation cross section σ. The electron flux profile *f*(*r*) at the sample surface is taken to be radially symmetric and can be obtained from Monte Carlo simulations of the electron–solid interaction. Under these conditions the radially symmetric rate equation reads [22]

[3]
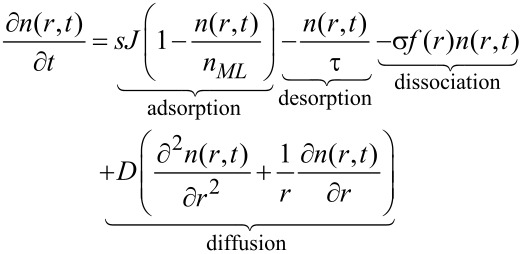


where *J* is the precursor flux modified by the sticking coefficient *s*. The local growth rate *R*(*r*) of the deposit, assuming the volume *V* for the nonvolatile dissociation product of an individual precursor molecule, is then obtained from

[4]
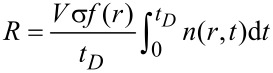


with *t_D_* denoting the beam dwell time.

Valuable insight can be obtained from the analytical solution of the rate equation, if the diffusion term is neglected. Depending on the diffusion constant this is a good approximation for short dwell times. Taking *f* = *f*(*r* = 0) as the electron flux at the beam center one obtains, after direct integration

[5]
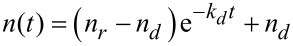


and consequently

[6]
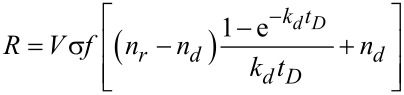


with the depletion rate *k_d_* defined as

[7]
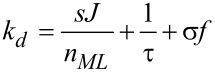


and the depleted adsorbate density *n_d_* = *sJ*/*k_d_*. The initial adsorbate density *n*(*t* = 0) was set to the adsorbate density after long times *n_r_* in the absence of the dissociation term. It is defined by the replenishment rate *k_r_* given by

[8]
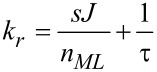


via the relation *n_r_* = *sJ*/*k_r_*.

**Figure 2:**
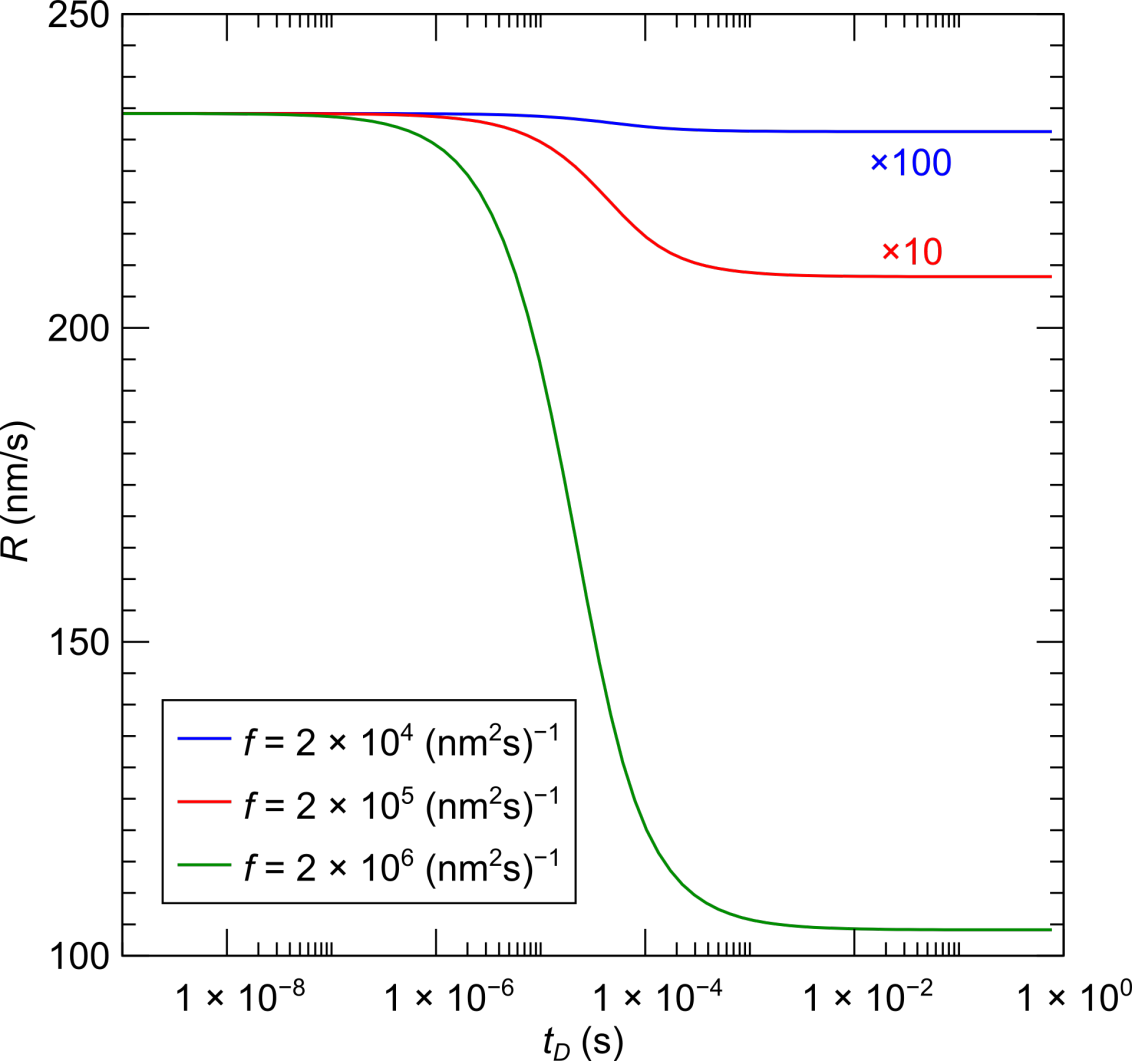
Single-species growth rate calculated for the precursor Me_3_Pt(IV)CpMe assuming three different electron-flux values as indicated. The flux values correspond to beam currents of approximately 0.1, 1 and 10 nA with a beam diameter of 20 nm. The model parameters σ = 2.2 × 10^−2^ nm^2^ [23] and τ = 29 μs [24] were used. The effective precursor flux was set to *sJ* = 1.5 × 10^3^ (nm^2^s)^−1^.

The important result obtained from this analysis is the generic shape of the deposit growth rate *R* as a function of the dwell time *t_D_*, as is shown in Figure 2. For the calculation, the a priori unknown model parameters σ and τ are needed. These can in fact be obtained from fitting of the dwell-time-dependent growth rates for different precursor flux settings *J* by using Equation 6, as e.g., detailed in Utke et al. [6]. Here parameters for the precursor Me_3_Pt(IV)CpMe have been used, as given in the figure caption.

Apparently the growth rate is proportional to the electron flux for very short dwell times, which is termed as the *reaction-rate-limited* (RRL) regime. For longer dwell times the precursor adsorbate becomes depleted as a consequence of the dissociation rate exceeding the replenishment rate. The resulting growth regime is *mass-transport-limited* (MTL). Since the characteristic dwell time for which, at a given precursor and electron flux, the crossover between the growth regimes takes place is precursor specific, an interesting FEBID-specific observation can be made with regard to using two (or more) adsorbate species. In this case, conditions can in principle be found under which, by varying the dwell time alone, the growth regimes for the precursors can be made to differ. As a consequence, under otherwise constant conditions the dwell time can be used as the decisive parameter to appreciably change the material composition. Firstly, this is of relevance in finding optimum process conditions for preventing the undesired inclusion of impurity adsorbates from the residual gas. In this case the residual gas adsorbate would act as a second precursor. Secondly, for the preparation of binary FEBID structures by using two or more functional precursors, a recipe for the fine-tuning of the material composition by variation of the dwell time can be devised. In the next subsection the continuum model will therefore be extended to a multicomponent variant.

#### Multicomponent extension of the continuum model

The extension of the model described above to the multicomponent case was first introduced by Lobo and Toth in order to describe simultaneous FEBID and focused electron beam induced etching (FEBIE) [25]. Etching can intentionally be induced by supplying a reactive precursor, such as XeF_2_. Bernau et al. adapted this model to describe the deposition process in the presence of a functional precursor and a typical hydrocarbon contaminant from the residual gas [26]. For two precursors the rate equations read

[9]
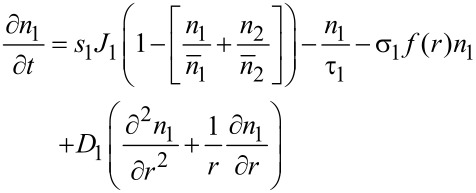


[10]
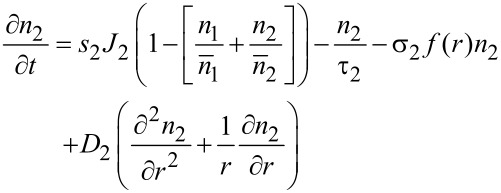


[11]
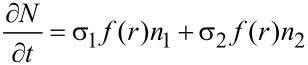


which we consider in the following in a simplified form without the diffusion term and taking again *f*(*r* = 0) = *f* as electron flux at the beam center

[12]
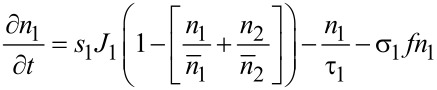


[13]
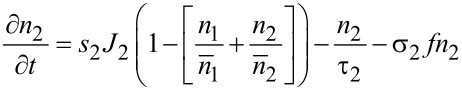


[14]
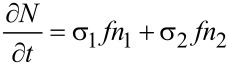


This system of coupled equations can be analytically solved and leads to

[15]
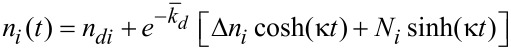


with *i* = 1, 2 using the following abbreviating definitions


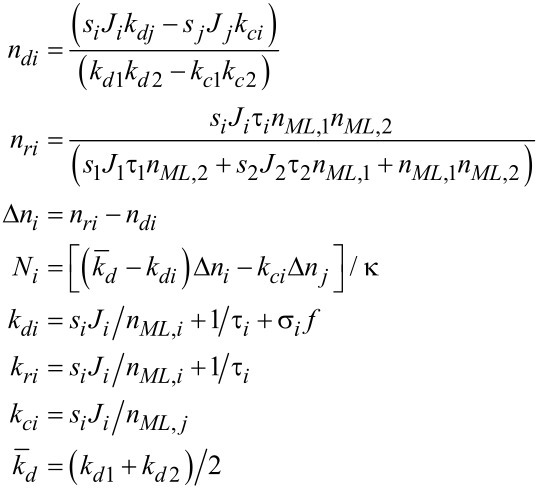


[16]
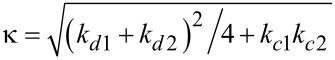


with (*i*, *j*) = (1, 2) or (*i*, *j*) = (2, 1), respectively. The initial conditions have again been set to the fully replenished state, i.e., *n_i_*(*t* = 0) = *n_ri_*.

An interesting piece of information to be obtained for these calculations is the expected yield ratio, that is the ratio of the dissociation rates per primary electron for the two precursors

[17]
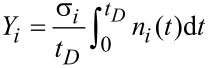


from which the yield ratio *Y*_1_/*Y*_2_ can be directly obtained.

**Figure 3:**
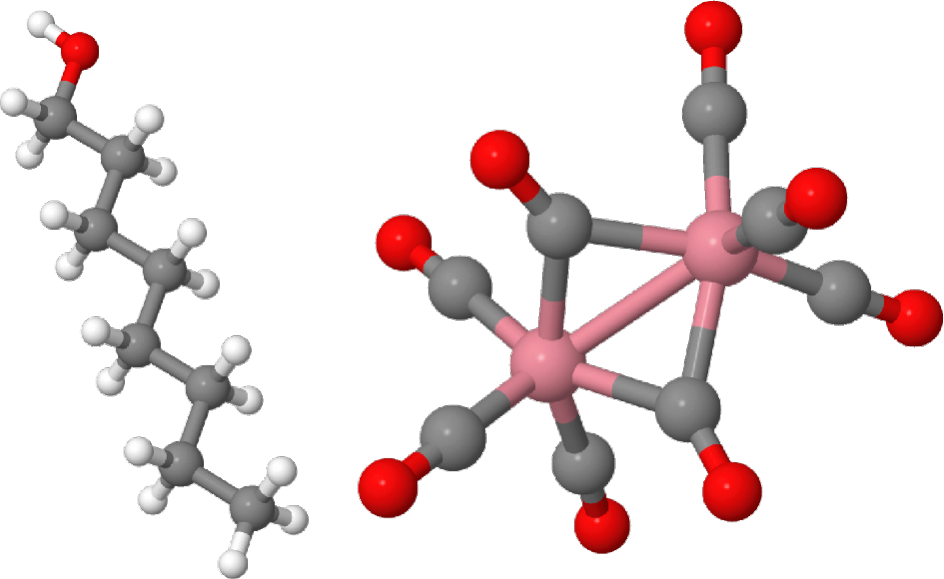
Molecular models of octanol (left) and Co_2_(CO)_8_ (right). Rendered using Jmol.

We now briefly review the results obtained by Bernau et al. [26] who studied the inclusion rate of carbon from the residual gas component octanol, C_8_H_17_OH, which is often found in high-vacuum systems that are pumped by diffusion pumps. At a background pressure of 1 × 10^−5^ mbar they estimated the effective impingement rate of octanol on the substrate surface to be *J*_2_ = 1.6 × 10^15^ (cm^2^s)^−1^. As the functional precursor, Co_2_(CO)_8_ was used at a flux of *J*_1_ = 1.5 × 10^17^ (cm^2^s)^−1^ (see Figure 3 for molecular models of the precursors). In independent octanol-free calibration measurements, the elemental composition was found to be Co_2_C_0.6_O_0.4_, i.e., a Co–Content of 66 atom %. Deposits from the residual gas contained carbon and oxygen in the ratio 8.5:1. The depositions were performed at a beam energy of 25 keV and a beam current of 1 nA. The FWHM of the electron beam was given as 70 nm, which translates to an electron flux of about 1.6 × 10^6^ (nm^2^s)^−1^. The monolayer densities were estimated from the dimensions of the intact molecules to be *n*_1_ = 2.6 nm^−2^ (Co_2_(CO)_8_) and *n*_2_ = 3.4 nm^−2^ (octanol).

**Figure 4:**
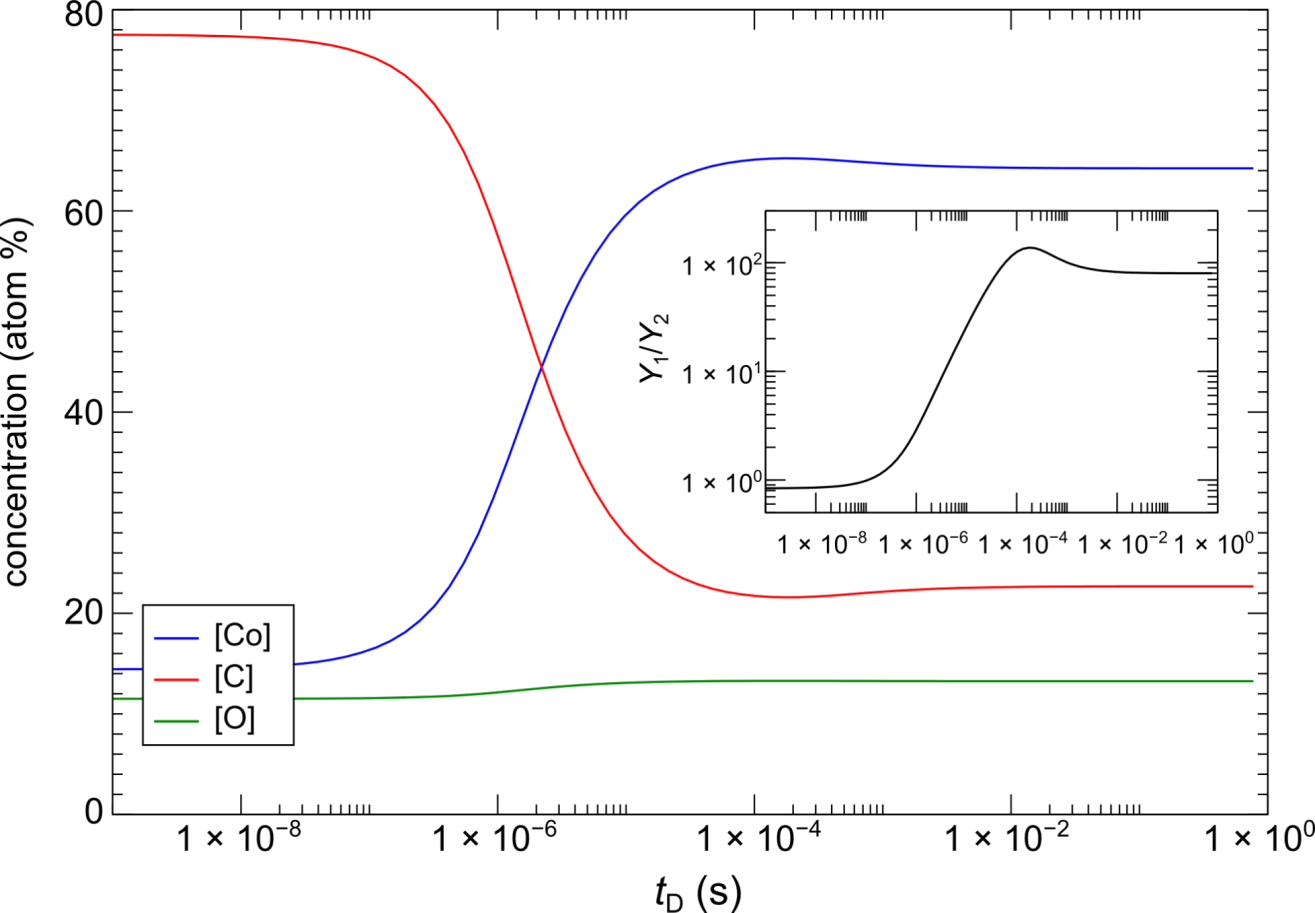
Simulation of concentration of different elements in FEBID structure under parallel use of Co_2_(CO)_8_ and octanol as precursors. The dissociation product of Co_2_(CO)_8_ is assumed to have the composition Co_2_C_0.6_O_0.4_, whereas for the octanol the composition C_8.5_O_1_ is assumed. Inset: Dissociation yield ratio for Co_2_(CO)_8_ and octanol from solving Equation 14 and using Equation 17 (abscissa units as in main graph). See text for details.

In order to determine the unknown quantities σ*_i_* and τ*_i_*, independent deposition experiments were performed under pure residual gas or quasi-pure Co_2_(CO)_8_ precursor conditions. The growth rate was in either case determined by measuring the height of the deposits by atomic force microscopy (AFM). From fitting of the obtained growth rates by using Equation 6 the following model parameters were obtained (see the supplementary information in Bernau et al. [26] for details): σ_1_ = 4.95 × 10^−3^ nm^2^, τ_1_ = 720 μs and σ_2_ = 2.1 nm^2^, τ_2_ = 190 μs. Employing these model parameters the yield ratios *Y*_1_/*Y*_2_ were calculated as a function of the dwell time. The results of this calculation are reproduced in Figure 4 (inset). After the composition of the deposits under single-precursor conditions (see above) was properly taken into account, this translates into the composition variation as function of dwell time as shown in Figure 4, which turned out to be in excellent agreement with the experimental observations.

In the next section binary FEBID materials will be discussed and the continuum growth model analysis of this section will be applied to the case of the parallel use of Co_2_(CO)_8_ and Me_3_Pt(IV)CpMe.

### Binary FEBID structures

The parallel use of two (or more) precursors in FEBID provides access to a whole new class of functional nanostructures. FEBID structures with tailored cooperative ground states, such as superconductivity and magnetism, can be envisioned. However, one has to keep in mind that the local growth rates in FEBID are high and that the beam-induced chemistry is presently neither well-understood nor well-controlled in most cases of single-precursor usage, not to mention precursor mixtures. As a consequence, due to strong kinetic limitations (large growth rate, reduced diffusion in the presence of two precursor adsorbates) a strong inclination to the formation of amorphous material can be expected. Nevertheless, in the case of combining an organometallic precursor with metal species that exhibit large mixing enthalpies and tend to form either alloys and intermetallic compounds, or that are continuously mixable, a crystallized product may be expected even under rapid growth conditions, such as in FEBID.

In this section two examples of binary FEBID will be discussed. So far, very little work has been done in this field. Che et al. reported on FEBID of FePt nanopillar structures by using Fe(CO)_5_ and Me_3_Pt(IV)CpMe as precursor gases in parallel [27]. The originally amorphous deposits were shown to crystallize into the L1_0_ “face-centered tetragonal” structure of FePt after an in situ annealing step at 600 °C. The magnetic analysis was performed by using off-axis magnetic holography in a transmission electron microscope (TEM) and provided evidence for the hard-magnetic nature of the FePt nanorods. Unfortunately, very little details concerning the FEBID growth parameters and precursor flux conditions were provided in this report. In particular, the elemental compositions under different precursor mixing ratios were not given, therefore any comparisons with the continuum-growth-model approach from the previous section are not possible.

The next section reports on binary FEBID focused on the fabrication of Pt–Si structures by employing the precursors Me_3_Pt(IV)CpMe and neopentasilane (Si_5_H_12_), the latter one being used for the first time in FEBID experiments [28]. Metal-silicides are highly relevant for metallization layers in integrated circuits. More importantly, the binary Pt–Si phase diagram shows several intermetallic phases, two of which are superconductors. It is thus worthwhile discussing the Pt–Si system in some more detail.

#### Pt–Si FEBID structures

As already alluded to in the last subsection the binary phase diagram of the Pt–Si system reveals two intermetallic compounds, which have a superconducting ground state. PtSi crystallizes in an orthorhombic structure with space group *Pnma*. Thin film studies on Pt layers on Si substrates show that PtSi forms at annealing temperatures above 600 °C via Si diffusion into the preformed Pt_2_Si phase, which has a body-centered tetragonal unit cell (space group *I*4/*mmm*) [29]. PtSi thin films become superconducting below *T*_c_ = 0.56 K [30]. A second Pt–Si phase of relevance for the present discussion is Pt_2_Si_3_, which is metastable and was found to form by annealing PtSi thin films of typically 30 nm thickness after Xe^+^ ion bombardment at 300 keV (integrated flux 1 × 10^15^ cm^−2^) [31]. The annealing was done at 400 °C for different time periods. The crystal structure of this metastable phase was resolved to be hexagonal, belonging to the space group *P*6/*mmc* [31]. Annealing at elevated temperatures (550 °C and above) leads to the destruction of the hexagonal phase under formation of PtSi and excess Si. A rather sharp superconducting transition was found for Pt_2_Si_3_ with an onset at 4.2 K [31].

**Figure 5:**
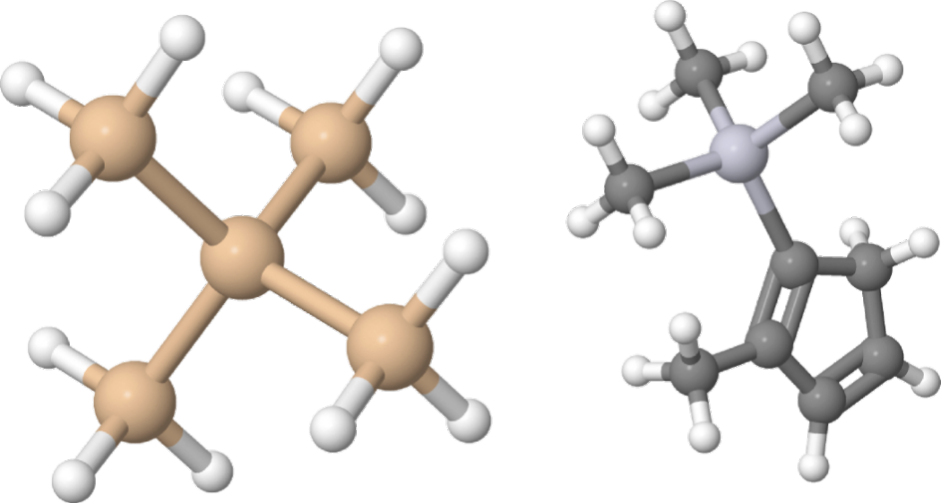
Molecular models of Si_5_H_12_ (left) and Me_3_Pt(IV)CpMe (right). Rendered using Jmol.

**Experimental**: We now turn to the results obtained in FEBID experiments by Winhold et al. employing Me_3_Pt(IV)CpMe and Si_5_H_12_ as precursors (see Figure 5) supplied by two independent gas injection systems [28]. In this work the liquid and pyrophoric precursor Si_5_H_12_ was used for the first time in FEBID as carbon-free source of Si. The experiments were performed in a dual-beam instrument (FIB/SEM, FEI Nova NanoLab 600) with a Schottky electron emitter. The beam voltage and current were 5 kV and 930 pA, respectively. The molecular flux ratio of the two precursor species was controlled by the distance of the Si_5_H_12_ gas injection capillary to the substrate surface (p-doped Si(100) with 300 nm thermally grown oxide), as well as a fine-dosing valve to control the Si_5_H_12_ molecular flux, keeping the Me_3_Pt(IV)CpMe molecular flux constant. Details concerning the absolute molecular flux values were not provided. The deposition parameters of 20 nm pitch and 1 μs dwell time were kept constant for all experiments. For the electronic transport measurements the structures were deposited between Au/Cr contacts previously defined by standard lithographic means. The temperature-dependent measurements were performed in a ^4^He cryostat with variable temperature insert.

**Figure 6:**
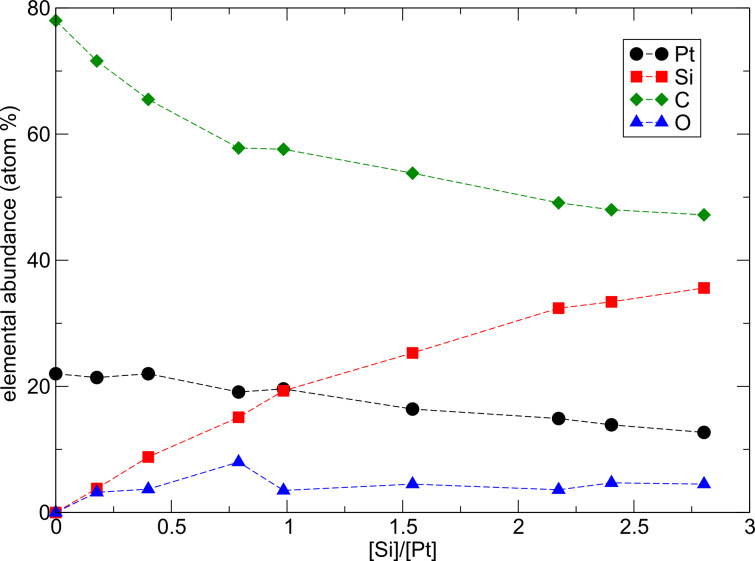
Elemental composition of various Pt–Si deposits as determined by EDX according to [28]. The data were taken after sample growth without a break of the vacuum.

In Figure 6 the results for the elemental composition of the deposits, as determined by energy dispersive X-ray analysis (EDX), are shown for nine samples. A priori it is not clear whether Si is preferentially included in the carbonaceous matrix or forms an alloy with Pt. From the EDX results several conclusions can be drawn, as was detailed in [28]. For low Si content a progressive decrease of the C content is observed accompanied by a parallel increase of Si and O. From this it may be concluded that Si is preferentially included in the C matrix and is partly oxidized in the presence of water and O_2_ from the residual gases in the electron microscope at a background pressure of about 6 × 10^−6^ mbar. This parallel growth of Si and O content ceases when a Si/Pt ratio of about 1 is reached. It is speculated that a substantial part of the Si content of the samples is now bound to the Pt, forming amorphous Pt–Si alloy structures. This assumption is to some degree corroborated by the results of transmission electron microscopy (TEM) investigations which show a progression from nanocrystalline fcc Pt particles in a carbon matrix for Si-free deposits, towards an amorphous structure of the granules. Since only a direct local probe, such as electron energy loss spectroscopy (EELS) in a TEM, would be able to unequivocally answer this question, we turn to some peculiarities observed in the transport-dependent conductivity of the FEBID samples.

**Figure 7:**
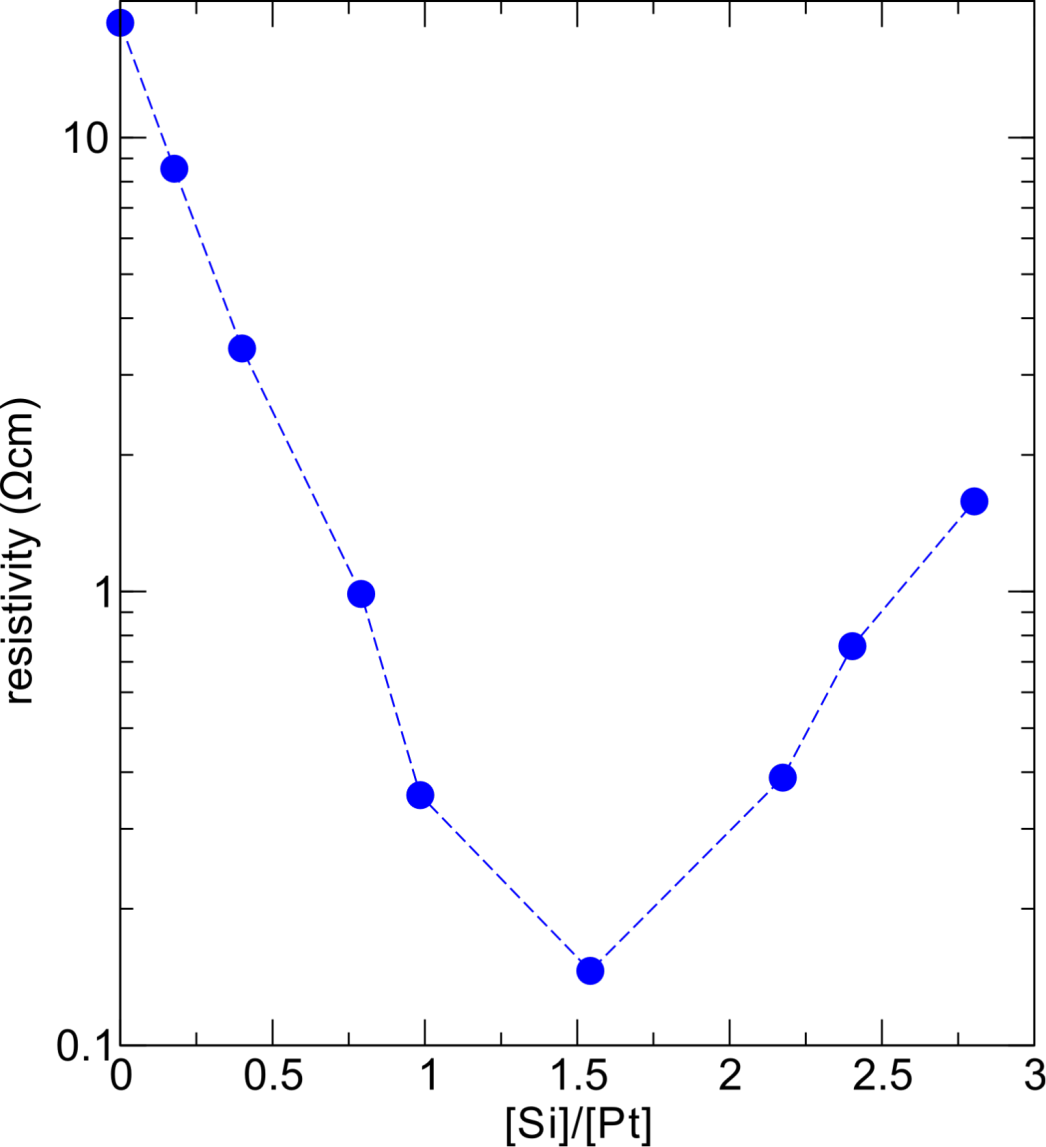
Dependence of the room temperature resistivity on the Si/Pt ratio in the FEBID samples according to [28].

**Figure 8:**
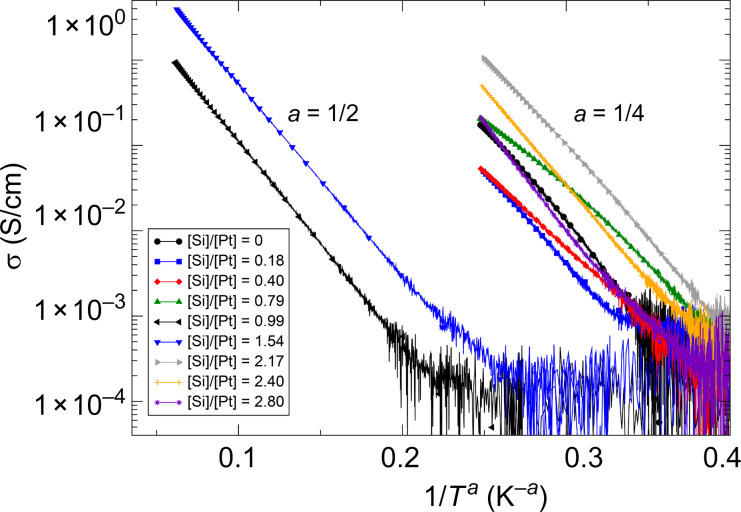
Temperature-dependent conductivity of the Pt–Si FEBID samples represented as ln σ vs *T*^−^*^a^* to facilitate comparison with VRH models according to Mott (*a* = 1/4, three spatial dimensions) and for correlated VRH (*a* = 1/2). The data are taken from Winhold et al. [28].

**Electronic transport properties**: Figure 7 shows the resistivity as a function of the Si/Pt ratio of the as-grown samples. Apparently, the resistivity drops with increasing Si content reaching a well-defined minimum at a composition close to Pt_2_Si_3_. The temperature-dependent conductivity, which is shown in Figure 8, indicates for this composition and also for the composition PtSi a special form of thermally activated transport, which is commonly associated to a variable-range hopping (VRH) conductance mechanism in the presence of electronic correlation effects [12], namely

[18]
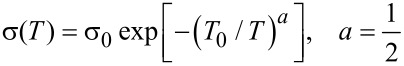


For all other samples either a VRH behavior in three dimensions (3-D) according to Mott (*a* = 1/4) [32] or some intermediate behavior is apparent.

The observed correlated VRH behavior observed for the samples with composition ratio [Si]/[Pt] = 1 and 3/2 indicates a granular electronic density of states of the FEBID samples. The minimal resistivity of the [Si]/[Pt] = 3/2 sample provides evidence that the intergranular tunnel-coupling strength is largest for this sample. On the other hand, from the TEM measurements no special microstructural feature, such as re-entrant crystallization of the granules, has been observed. It thus remains an unresolved issue, whether the granular electronic density of states is indeed indicative of the formation of an amorphous precursor of the metastable, hexagonal and superconducting Pt_2_Si_3_ phase. Unpublished results of one of us (M. W.) on the low-temperature resistance of FEBID samples with a composition close to [Si]/[Pt] = 3/2, which have been subjected to an extended post-growth electron irradiation treatment, show the onset of superconducting correlations below 4.2 K at large bias current. Future research will have to show whether this is the result of local crystallization towards the Pt_2_Si_3_-phase caused by the high dissipation levels under large current bias. For details concerning the electronic transport properties of nanogranular FEBID structures the reader is referred to the next section.

#### Co–Pt FEBID structures

As a second example of a binary FEBID experiment recent results on the Co–Pt system are reviewed [33]. The binary phase diagram of Co–Pt features several ferromagnetic intermetallic compounds. The most prominent of these is the L1_0_ phase of CoPt, which has a face-centered tetragonal structure and is hard magnetic at room temperature [34]. Without any doubt FEBID holds great promise to become an important fabrication technique for magnetic nanostructures for micromagnetic studies, such as in the area of artificial spin-ice systems [35] or dipolar coupling effects [36]. Several interesting investigations on the growth and magnetic properties of Co–C deposits employing the precursor have been published in recent years [13,37–42]. Co–C deposits have also been used in recent experiments on the guided motion of vortices in the Shubnikov phase of epitaxial Nb thin films [43–46]. Two issues have to be considered here. Firstly, the precursor is relatively unstable and dissociates, in particular under vacuum conditions, via the intermediate tetracobalt dodecarbonyl, into Co and carbon monoxide. Combined experimental and theoretical research has furthermore found clear indications that this precursor spontaneously dissociates on non-hydroxylated SiO_2_ surfaces, i.e., on substrate surfaces often used in FEBID [20]. In this same research the catalytic decomposition of Co_2_(CO)_8_ on previously formed Co structures has also been experimentally demonstrated. Great care has therefore to be taken when this precursor in used. Secondly, FEBID structures from Co_2_(CO)_8_ can have a metal content of more than 95 atom % and show temperature-dependent transport properties reminiscent of dirty Co thin films in combination with soft-magnetic behavior at room temperature [13]. It would be desirable to also have access to hard-magnetic structures via the FEBID route. In this regard CoPt in the L1_0_ phase represents an excellent choice.

**Experimental**: The experiments were performed in a dual-beam microscope with Schottky electron emitter (FIB/SEM, FEI Nova NanoLab 600) at 5 keV beam energy and 1.6 nA current. The writing parameters were 20 nm pitch and 1 μs dwell time. p-Doped Si (100) substrates with 200 nm of thermally grown oxide were used. The structures were deposited between Au/Cr contacts previously defined by standard lithographic means. The molecular flux ratios of the employed precursors Co_2_(CO)_8_ (at 28 °C) and Me_3_Pt(IV)CpMe (at 52 °C) were adjusted by varying the distance between the Co_2_(CO)_8_ injector’s capillary exit and the substrate surface at a beam focus between 7 and 26 mm while keeping the Me_3_Pt(IV)CpMe injector’s capillary exit at a fixed distance of 32 mm. No absolute values for the molecular flux were provided in [33]. The transport measurements were performed in a variable-temperature insert mounted in a ^4^He cryostat with a superconducting solenoid. Two series of three samples were grown close to the 1:1 composition ratio of Co and Pt. One sample set was treated by post-growth electron irradiation by using the same beam parameters as in the deposition experiments. The elemental composition of the samples was determined by EDX. In Table 2 relevant information concerning the samples is compiled for ease of reference. For more details the reader is referred to [33].

**Table 2:** Sample composition and Co_2_(CO)_8_ injector distance *s*_Co_ for Co–Pt samples. Samples A, B and C are as-grown. Samples A', B' and C' are subject to a post-growth electron-irradiated treatment with doses of 10.58, 7.02 and 14.24 μC/μm^2^, respectively. For all depositions the Me_3_Pt(IV)CpMe injector’s capillary distance to the substrate surface at the focus of the electron beam was kept constant at *s*_Pt_ = 32 mm. Table data reproduced from [33].

sample	[Co] (atom %)	[Pt] (atom %)	[C] (atom %)	[O] (atom %)	[Co]/[Pt]	*s*_Co_ (mm)

A	16.9	12.5	60.2	10.4	1.35	7
B	13.4	13.9	60.6	12.1	0.96	10
C	8.9	14.8	62.0	14.3	0.6	26
A'	21.3	16.5	40.2	22.0	1.29	7
B'	15.7	18.2	47.4	18.7	0.86	10
C'	13.5	22.4	37.7	26.4	0.6	26

**Figure 9:**
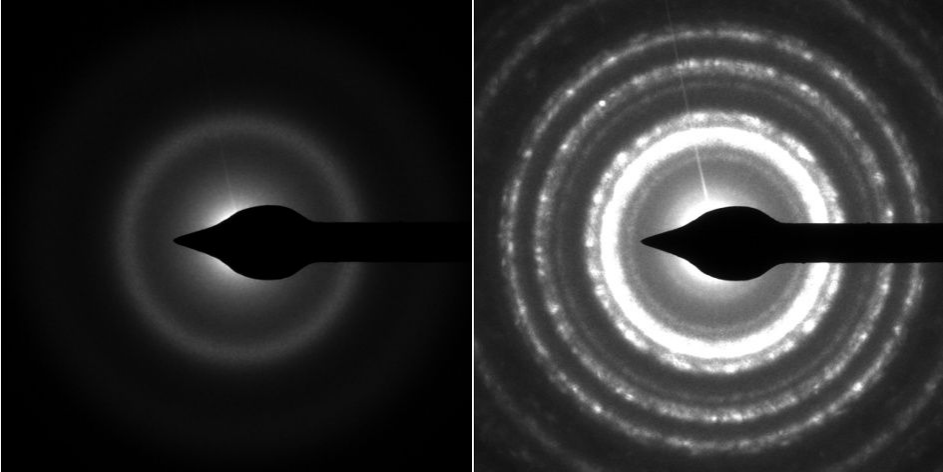
TEM electron diffraction pattern of samples on carbon membrane before (left) and after (right) postgrowth electron irradiation. The phase transformation from an amorphous to a crystalline state of the Co–Pt grains is apparent. See text for details. Images reproduced from Porrati et al. [33].

**Microstructural characterization**: TEM investigations (FEI Tecnai F20 at 200 kV beam voltage) were performed on samples prepared in independent experiments on 30 nm thick carbon membranes. The sample composition for the as-grown sample as well as the sample for post-growth electron irradiation (dose 8.64 μC/μm^2^) was tuned to that of sample B, i.e., close to a [Co]/[Pt]-ratio of 1. From bright-field imaging a nanogranular structure was deduced with Co–Pt grains embedded in an amorphous, carbonaceous matrix. Figure 9 shows the diffraction images of the as-grown and post-growth irradiated sample for comparison. Apparently, the diffraction contrast for the as-grown sample is weak, indicating a largely amorphous state of the Co–Pt grains. This changed appreciably after the postgrowth electron-irradiation treatment. A multitude of well-defined diffraction rings formed, which can be unequivocally attributed to the L1_0_ intermetallic phase of CoPt, as detailed in Porrati et al. [33].

**Figure 10:**
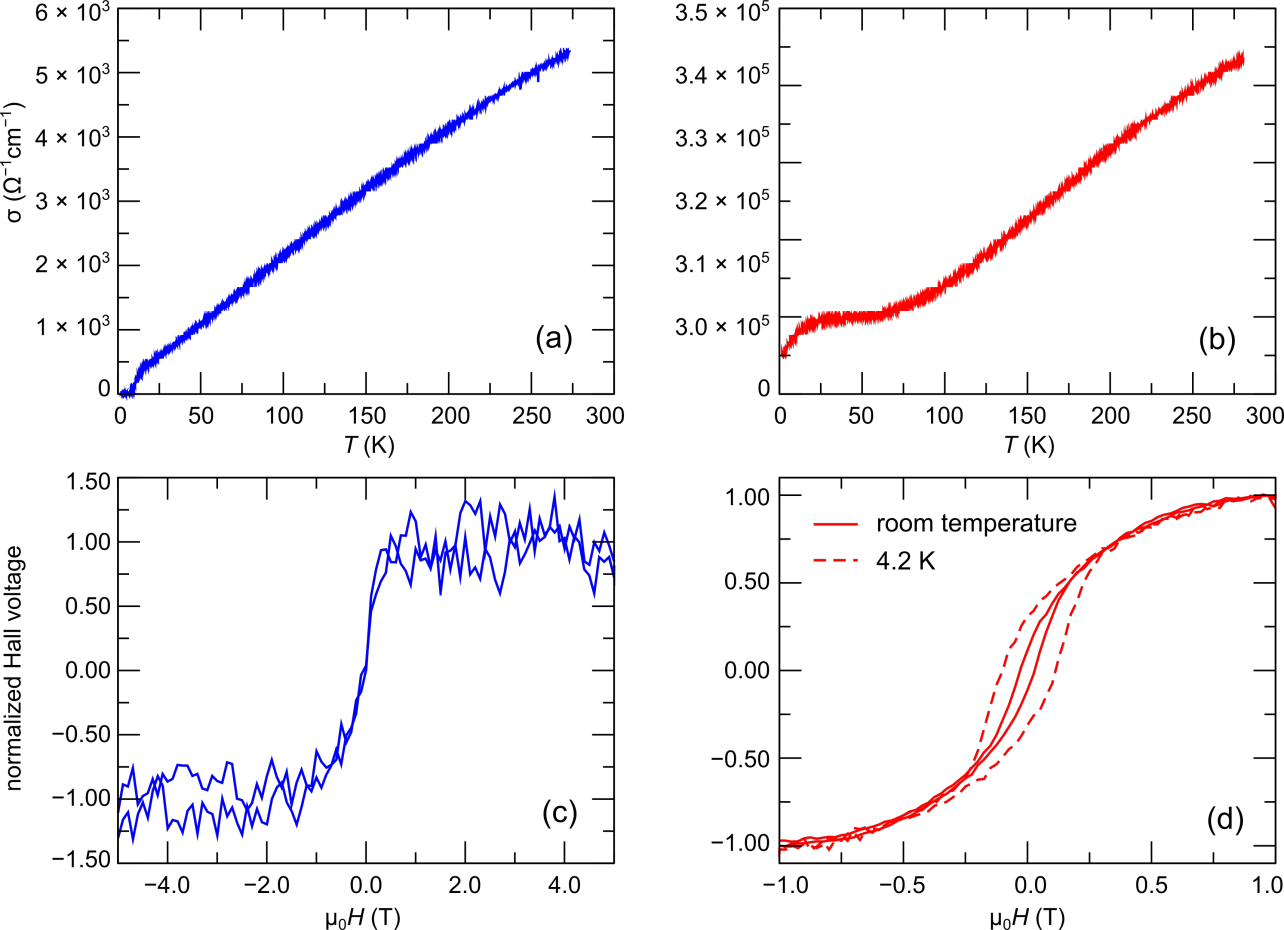
Temperature-dependent conductivity and Hall effect as a function of the applied magnetic field for samples B (a,c) and B' (b,d). The Hall data shown in (c) were taken at 228 K. Adapted from [33].

**Magnetic and transport properties**: Selected results from the electronic transport measurements comprising the temperature-dependent conductivity and the magnetic field dependence of the Hall voltage are shown in Figure 10 for sample B (Figure 10a and Figure 10c) and B' (Figure 10b and Figure 10d). The as-grown sample exhibits a roughly linear temperature dependence of the conductivity down to about 12 K, which is followed by a quite sudden drop to a very small conductance level. The anomalous Hall effect, indicative of the Hall contribution proportional to the sample’s magnetization, shows superparamagnetic behavior at room temperature. Data at low temperature could not be taken due to noise issues. From these observations, and in particular with regard to the sudden drop in conductance below 12 K, a glassy transition from a superparamagnetic state to a super-spin-glass [47] state may be assumed. However, further work on the low-temperature magnetic state of these deposits is needed before a definite statement can be made. More importantly, the conductivity of the postgrowth electron irradiated sample shows an increase by about two orders of magnitude. The conductivity levels off below 50 K and shows only a small residue of the conductance drop at 12 K. The Hall data indicate now a ferromagnetic state at room temperature with increasing coercive field as the sample is cooled to low temperatures. This indicates that the phase transformation from an amorphous to the ordered L1_0_ phase is accompanied by a corresponding phase transition from a superparamagnetic to a moderately hard ferromagnetic state. The overall magnetic properties of these samples depend strongly on the magnetic intergrain interaction, which has tunnel-exchange and dipolar contributions. Since the coupling strength is tunable, as indicated by the strong increase of the conductivity after post-growth irradiation, FEBID of nanogranular Co–Pt systems provides a particularly elegant pathway to sample preparation for the study of different collective magnetic states.

**Modeling within the multicomponent continuum scenario**: We now turn to a more in-depth analysis of the composition of the deposits obtained from the parallel dissociation of the two precursors. The analysis is mainly based on the multicomponent continuum growth model of FEBID reviewed in the first section. Such an analysis has not been done so far and may provide some leads for future work on this binary system with regard to the fine-tuning of the elemental composition.

The compositional analysis from EDX measurements yields the relative fractions or concentrations [X] of the constituent elements in the deposits. From the continuum model analysis, on the other hand, the individual yields *Y_i_* of the nonvolatile dissociation products for each precursor, as given by Equation 17, are obtained. Within the model assumptions of noninteracting precursor fragments, i.e., under the assumption that no secondary chemical reactions are taking place between volatile dissociation fragments, the concentration ratios [X]/[Z] of two elements in the binary deposit can be calculated from the yields as follows

[19]
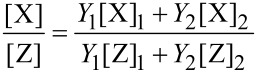


where [X]*_i_* and [Z]*_i_* (*i* = 1, 2) represent the concentration of the respective elements in the nonvolatile dissociation products of each of the two precursors individually. In turn, this allows for determination of the yield ratios from the found elemental composition ratios

[20]
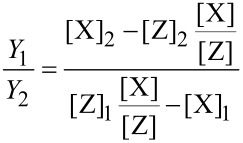


In Table 3 the element concentrations of deposits formed from the individual precursors are compiled for the experimental conditions specified in Porrati et al. [33]. From these the yield ratio *Y*_1_/*Y*_2_ = 0.282 is obtained by using the elemental concentration ratio for sample B, [Co]/[Pt] = 0.96.

**Table 3:** Composition of nonvolatile dissociation products from the individual precursors Co_2_(CO)_8_ and Me_3_Pt(IV)CpMe on Si/SiO_2_ substrates at 5 keV beam energy, 1.6 nA beam current (measured at Faraday cup), 20 nm pitch and 1 μs dwell time.

	[Co]*_i_*	[Pt]*_i_*	[C]*_i_*	[O]*_i_*

dissociation product precursor 1: Co_2_(CO)_8_	0.75	0.0	0.17	0.08
dissociation product precursor 2: Me_3_Pt(IV)CpMe	0.0	0.22	0.78	0.0

The expected elemental concentrations of C and O can now be predicted within the assumptions of the continuum model (index *cm*) by using Equation 19. One obtains ([C]/[O])*_cm_* = 36.6 and ([C] + [O])/([Co] + [Pt])*_cm_* = 1.97. From the experimentally determined elemental composition one derives [C]/[O] = 5.0 and ([C] + [O])/([Co] + [Pt]) = 2.66 (see data in Table 2). This allows directly for a qualitative assessment of the applicability of the continuum model for this binary system. The neglect of interfragment reactions in the model leads to an underestimate of the abundance of the nonmetallic precursor fragments in the deposits. Apparently, the parallel dissociation of the oxygen-free Me_3_Pt(IV)CpMe and oxygen-containing Co_2_(CO)_8_ precursor leads to an enhanced inclusion of oxygen. In parallel, the overall concentration of the nonmetallic components increases. It may be speculated that secondary reactions between the volatile precursor fragments, e.g., oligomerization, lead to the formation of less-volatile organic species, which are eventually included in the deposits. This simple analysis makes it quite clear that a more detailed understanding of the fragmentation and reaction pathways is needed for a thorough understanding of the process of deposit formation. In principle, the continuum model can be extended to include secondary reactions as long as the corresponding reaction rate parameters can be deduced from independent experiments. Future work on the development of a better understanding of the FEBID process will have to make use of surface-science techniques under well-controlled experimental conditions, in particular ultrahigh vacuum, to allow for a detailed analysis of the reaction mechanisms. For selected examples this has already started [17].

**Figure 11:**
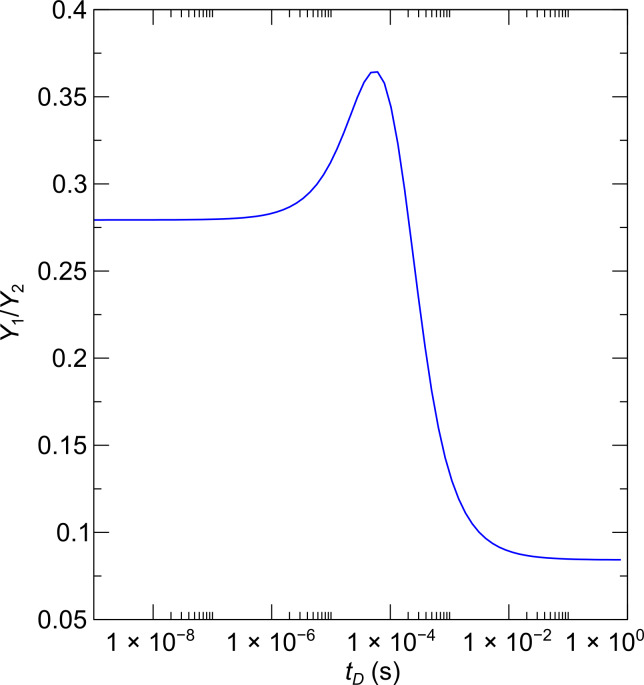
Dependence of the yield ratio for the precursors Co_2_(CO)_8_ and Me_3_Pt(IV)CpMe on the dwell time within the multicomponent continuum model of FEBID. See text for details.

Although the predictive power of the multicomponent continuum model of FEBID is limited, it can nevertheless provide useful information with regard to the dependence of the sample composition of the dwell time at fixed molecular fluxes. To show this, the dependence of the yield ratio *Y*_1_/*Y*_2_ on the dwell time has been calculated by using Equation 15 and Equation 17 with the Co_2_(CO)_8_ precursor parameters introduced in the first section for a reduced molecular flux value of 75 (nm^2^s)^−1^ owing to the larger capillary distance of 10 mm. The electron flux was set to 1.6 × 10^6^ (nm^2^s)^−1^. The model parameters for Me_3_Pt(IV)CpMe were extracted from the literature, namely σ_2_ = 2.2 × 10^−2^ nm^2^ [23], τ_2_ = 29 μs [24] and 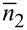 = 2.0 nm^−2^ [24]. The precursor flux for Me_3_Pt(IV)CpMe was set to 54 (nm^2^s)^−1^ so that the calculated yield ratio corresponded to the value of 0.283 derived for sample B previously. Figure 11 shows that the yield ratio can be tuned to a large degree by simply changing the dwell time. In particular, within the dwell time range of 1 μs to 10 ms the yield ratio can be changed by a factor of three. This should allow for a very fine tuning of the [Co] versus [Pt] concentration in FEBID experiments at otherwise fixed deposition conditions. An analogous behavior is expected for other binary systems.

### Nanogranular FEBID structures

On a very general level FEBID structures can be classified as disordered electronic materials. In between the extreme cases of fully amorphous deposits and polycrystalline structures with some degree of defects falls the class of nanogranular systems. They consist of nanocrystallites embedded into a carbon-rich dielectric matrix, which are subject to an intergranular electronic coupling due to a finite tunneling probability between the crystallites or grains. The binary systems Pt–Si and Pt–Co discussed previously fall into this class. For nanogranular materials the semiclassical approach of Boltzmann transport theory is not applicable since disorder does not simply cause scattering but must be included in the theoretical analysis from the beginning. A recent theoretical review on the electronic transport properties of granular metals can be found in [12].

The electrical transport within the metallic grains can be considered diffusive due to intragrain and surface scattering. Despite this scattering there is a well-defined and important intragrain energy scale, which is the mean spacing δ between the one-electron levels close to the chemical potential of the grain. It is given by δ = 1/*N_F_V*, where *V* 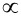 *r*^3^ is the grain volume (*r*: grain radius) and *N_F_* denotes the density of states at the chemical potential. For typical grain sizes in FEBID structures with a diameter of a few nanometers, δ/*k*_B_ (*k*_B_: Boltzmann constant) is of the order of 1 K for metallic grains with a density of states on the order of 1 (eVnm^3^)^−1^. From this rough estimate, one can directly conclude that quantum size effects due to the discrete energy levels can only become relevant at very low temperatures.

The electronic (transport) properties of granular metals depend sensitively on the average tunnel conductance *G* between neighboring grains, which is commonly expressed as the dimensionless quantity *g* = *G*/(2*e*^2^/*h*), i.e., normalized to the conductance quantum. Metallic behavior will be observed, if *g* surpasses a critical coupling strength *g_c_* ≈ 1. Samples with *g* < *g_c_* show insulating behavior, i.e., zero conductance as *T* → 0. The notion *metallic* does not necessarily imply a positive temperature coefficient of the resistance but merely means a finite conductivity as *T* approaches 0. The formal condition for a material to qualify as a granular metal is that the intergrain coupling strength *g* is much smaller than the normalized conductance *g*_0_ inside a grain.

Due to the tunnel-coupling between the grains the one-electron energy levels at the chemical potential are broadened. This effect is expressed by the broadening parameter Γ = *g*δ. Another important parameter is the single-grain Coulomb charging energy *E_C_* = *e*^2^/2*C* where *C* 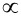 *r* is the capacitance of the grain. *E_C_* is equal to the change in electrostatic energy of the grain when one electron is added or removed. For insulating samples charge transport is suppressed at low temperatures due to this charging energy. The average level spacing δ can become larger than the charging energy only for very small grains. For FEBID samples, however, typical grain sizes are in the range of several nanometers, and the assumption *E_C_* > > δ is well justified.

### Transport theory of granular metals

A detailed review of the theory necessary to describe the transport properties of granular metals goes far beyond the present review, and the reader is referred to Beloborodov et al. [12]. Nevertheless, a short account is given to provide a framework for the following discussion of the experimental findings on nanogranular FEBID structures prepared by using the precursor Me_3_Pt(IV)CpMe.

Neglecting spin, which is of no relevance for nonmagnetic granular metals, the Hamiltonian has three components:

[21]
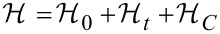


where 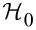 comprises the intragrain kinetic and potential energies

[22]
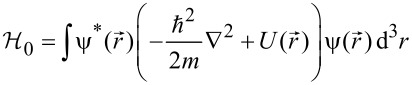


and 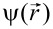 denotes the field operator representing the electron field. 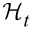 describes the tunneling between the grains

[23]
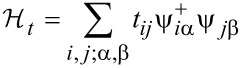


with (*α*, *β*) indexing the internal energy levels of the coupled grains with indices (*i*, *j*). The Coulomb charging energy is expressed through the capacitive coupling *C_ij_* between the grains

[24]
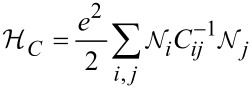



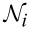
 denotes the electron number operator as the difference from the charge neutral state with *N* electrons per grain

[25]
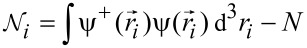


By means of field-theoretical methods (bosonization techniques, perturbation theory in the strong-coupling regime *g* ≥ 1), and under the assumption of a regular lattice of identical grains in one, two or three spatial dimensions (*d* = 1,2,3), the following results for the temperature-dependent conductivity are obtained.

In the *metallic state* a universal logarithmic conductivity correction is obtained that saturates for *k*_B_*T* < Γ. For *k*_B_*T* < Γ a dimension-dependent higher-order correction indicates the development of a coherent transport regime, which is denoted as a granular Fermi liquid [48]

[26]
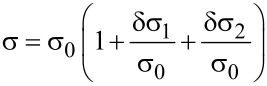


with

[27]
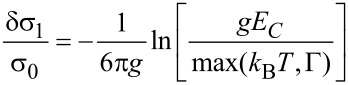


and

[28]
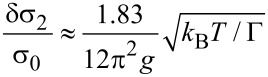


The expressions are here only given for the case *d* = 3, as this is of relevance for the analysis of the transport properties of FEBID structures prepared so far.

In the *insulating regime* the theory predicts a hard energy gap 
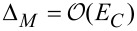
 resulting in an Arrhenius-like conductivity

[29]
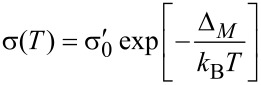


However, the experimental findings for granular metals in the insulating regime indicate a different activated behavior of the form

[30]
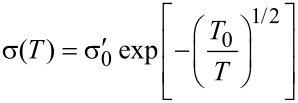


which we denote as *correlated VRH*. Beloborodov et al. [49] provided a theoretical explanation by observing that inelastic (at higher temperature) and elastic (at low temperature) cotunneling of electrons through many grains, in conjunction with random chemical potential fluctuations in the grains, caused by charged impurities in the matrix and at surfaces, will smear out the hard energy gap and lead to the observed correlated VRH.

**Figure 12:**
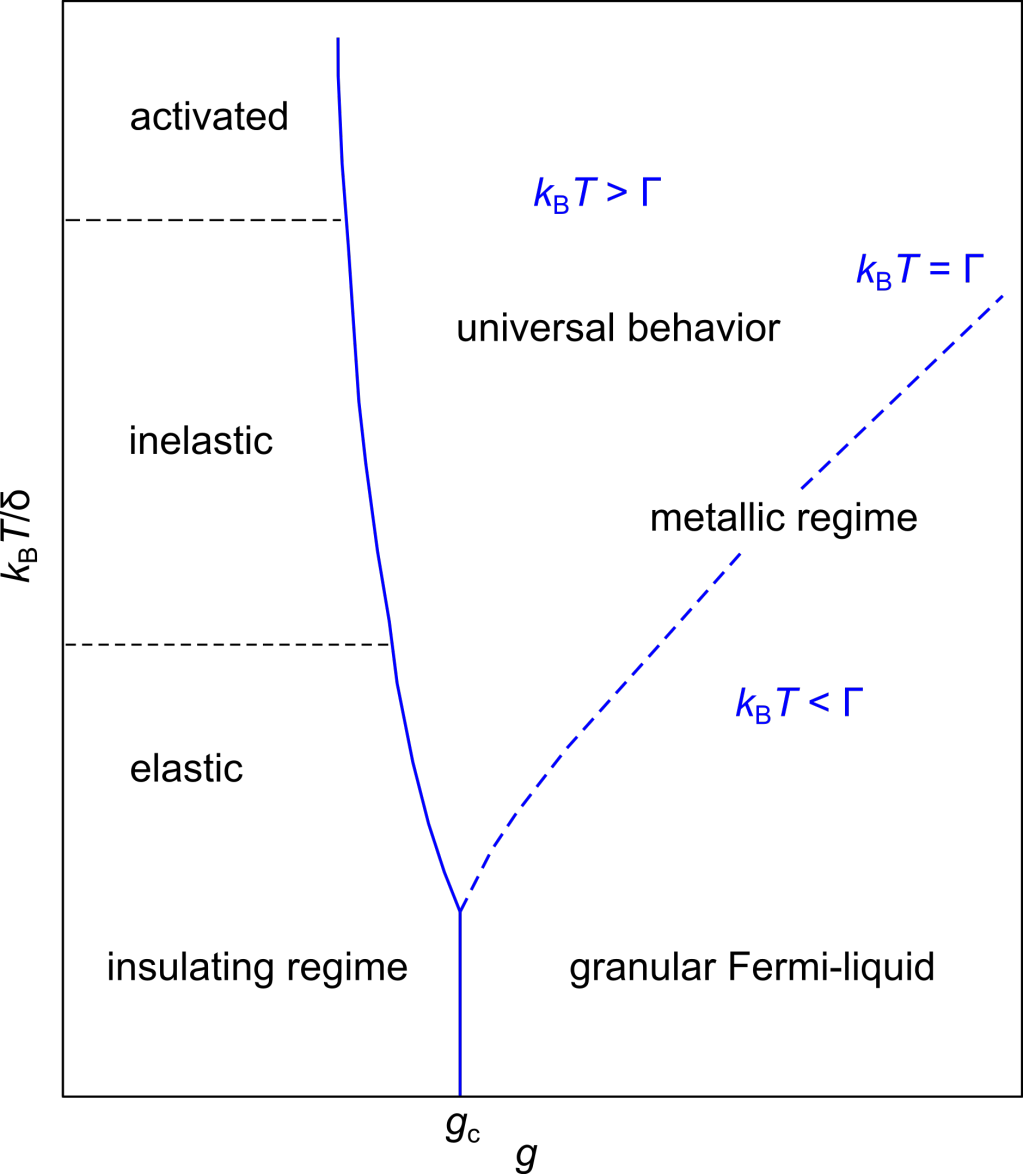
Phase diagram of the transport regimes of granular metals. In the insulating regime for *g* < *g_c_* thermally activated transport is observed of the Arrhenius type at elevated temperatures, which crosses over to correlated VRH due to inelastic or elastic cotunneling in the presence of potential disorder in the grains. In the metallic regime for *g* > *g_c_* a universal logarithmic correction for *k*_B_*T* > Γ is expected, which saturates at lower temperature as coherent transport develops (granular Fermi liquid). Adapted from [48].

The results of this theoretical analysis can be conveniently compiled into a phase diagram of the transport regimes of granular metals [48], which is shown in Figure 12.

#### Tunable granular metals prepared by FEBID

Initial experiments addressing in particular the transport properties on the metallic side of the insulator-to-metal transition of FEBID structures were performed on the W–C–O-system prepared from the precursor W(CO)_6_ [50]. In this case, the metal content was increased by carefully tuning the beam parameters. Since changes of the metal content are in general associated with corresponding changes in the microstructure, the interpretation of transport properties is not simple. Recent experimental findings in the transport properties of Pt–C FEBID structures prepared with the precursor Me_3_Pt(IV)CpMe allow for a particularly elegant way of testing the theoretical predictions presented in the previous subsection. In experiments on optimizing Pt–C FEBID structures for strain-sensor element applications (see next section) it was found that a strong increase of the conductivity by up to four orders of magnitude can be obtained by post-growth electron irradiation of the deposits [51]. Subsequent work identified the dominant reason for this apparent increase of the intergrain tunnel-coupling strength *g* to be caused by a microstructural change of the prevailing hybridization state of the C atoms in the matrix from amorphous carbon to nanocrystalline graphite [52]. This conclusion was drawn from the observed peak shifts and changes of the spectral weight of C-specific vibrational eigenmodes in Raman spectra of deposits that were subject to different post-growth electron irradiation doses. In follow-up work an optimized post-growth irradiation protocol was described that leads to an equally strong conductivity increase for shorter irradiation times [53]. In this subsection the focus is on the analysis of the temperature-dependent conductivity of these Pt–C deposits, which cover the full range from insulating to metallic behavior, i.e., *g* << 1 to *g* > *g_c_* ≈ 1.

**Experimental details**: The experiments were performed in a dual-beam microscope (FEI Nova NanoLab 600) with Schottky emitter. The precursor Me_3_Pt(IV)CpMe was heated to a temperature of 52 °C. A series of samples with a lateral size of 5 × 1 μm^2^ were prepared between prefabricated Au/Cr electrodes under identical conditions of 5 keV, 1.6 nA (measured at Faraday cup), 20 nm pitch and 1 μs dwell time on a p-doped Si (100) substrate with 100 nm thermally grown oxide held at room temperature. In the as-grown state the samples had a thickness of 80 nm. After growth, the samples were subjected to a post-growth electron irradiation treatment of different duration employing the same beam parameters as used for the deposition. During the irradiation treatment the sample height showed a rapid drop by approximately 20% within the first 20 min. This was followed by a gradual thickness reduction over several 100 min down to approximately 55% of the original thickness for the samples subject to a long-term irradiation treatment [52]. This apparent volume loss is thought to be caused by the dissociation of residual precursor fragments embedded in the deposits during growth [53] and the partial loss of carbon due to electron-stimulated reaction with residual water to carbon monoxide [52].

**Figure 13:**
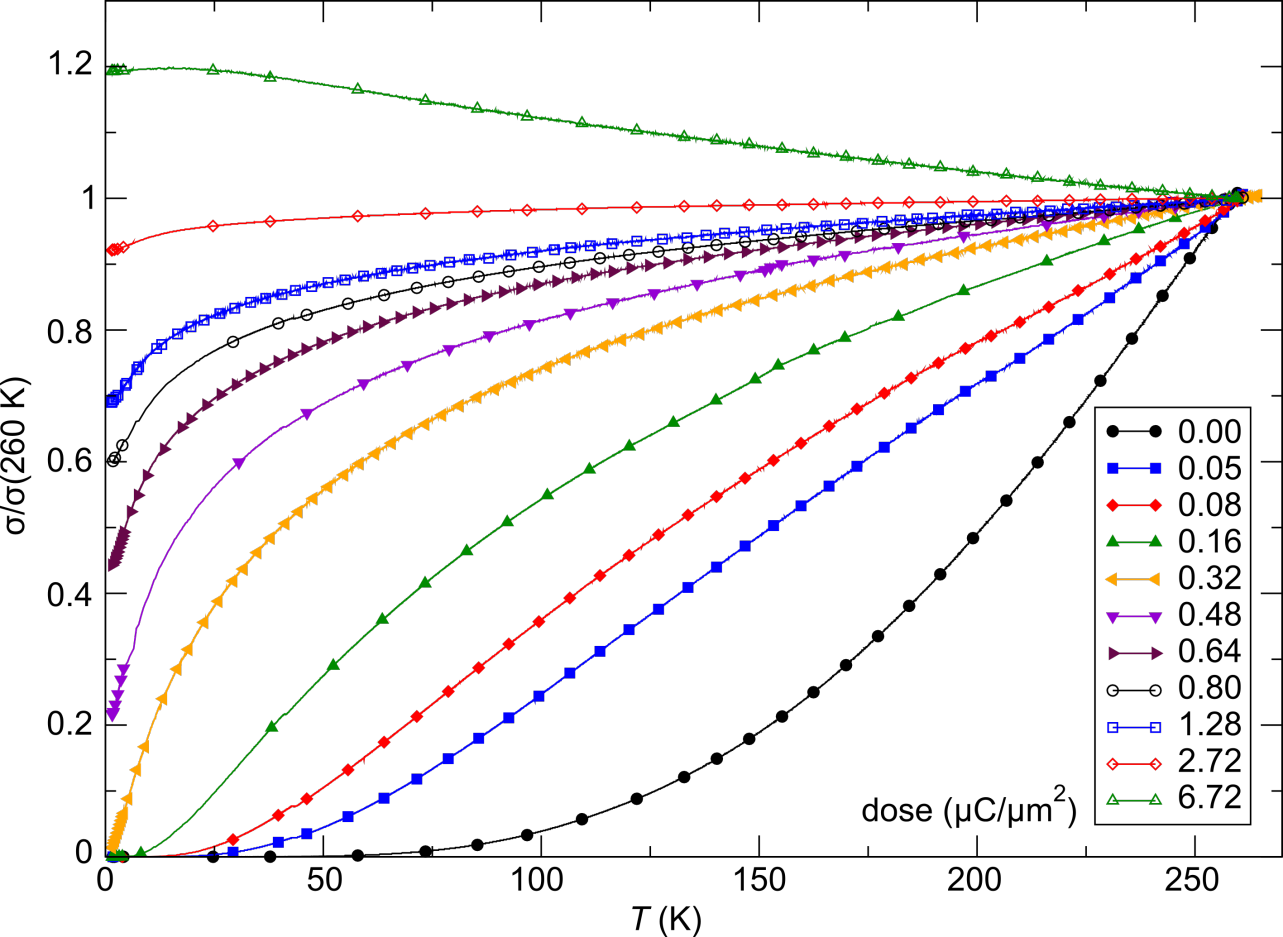
Temperature-dependent conductivity of Pt–C FEBID structures that have been exposed to different post-growth electron irradiation doses as indicated. See text for details. Adapted from [52].

**Temperature-dependent conductivity**: Figure 13 shows an overview of the temperature-dependent conductivity of the samples exposed to different irradiation times as indicated. It is directly apparent that the Pt–C system can be finely tuned through a insulator-to-metal transition. The highly reproducible growth characteristic represents one particular advantage of this system. This ensures that under nominally identical conditions samples of very similar transport properties can be obtained. The irradiation-induced increase of the conductivity of up to four orders of magnitude as specified in Porrati et al. [52] is not apparent from the normalized representation.

**Figure 14:**
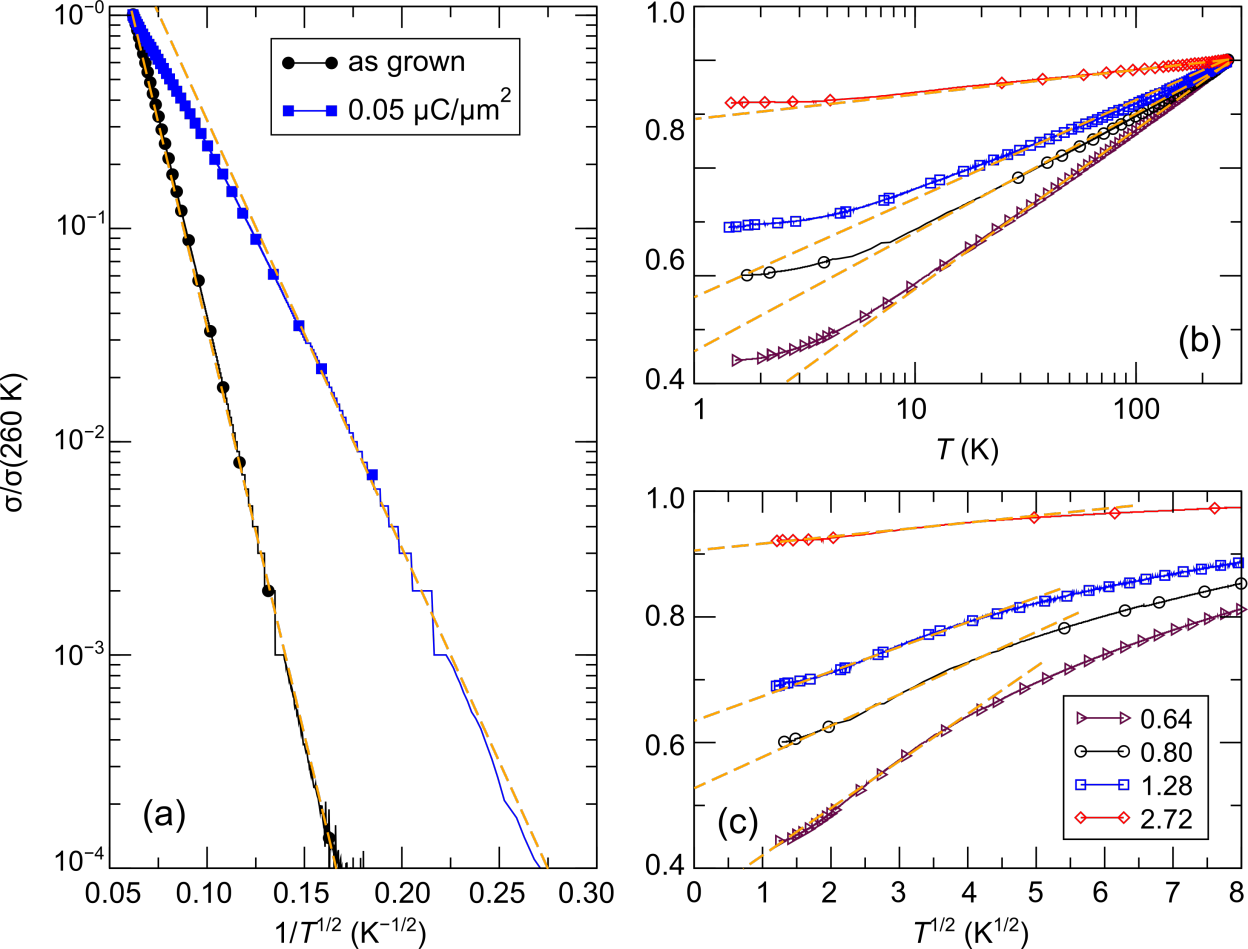
Temperature-dependent conductivity of Pt–C FEBID structures that have been exposed to different post-growth electron irradiation doses as indicated. (a) As-grown briefly irradiated samples show thermally activated behavior following the correlated VRH scenario. (b) Long-term irradiated samples reveal a logarithmic temperature dependence of the conductivity in accordance with Equation 27. Saturation of this behavior is observed below about 15 K. (c) Samples in the metallic regime at low temperature show indications for a crossover to a 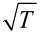-dependence of the conductivity. The straight dashed orange lines in the plots are meant to facilitate a judgement of the quality of the temperature dependence of the conductivity as predicted by theory. See text for details. Adapted from [54].

Further analysis reveals that the as-grown sample shows correlated variable-range hopping according to Equation 30 over the complete measured temperature range. The same holds true for samples subject to small irradiation doses in the low-temperature region. As room temperature is approached a deviation from correlated VRH is observed, which may indicate the expected cross-over to simple Arrhenius behavior. However, further temperature-dependent measurements above room temperature are needed to clarify this point. Figure 14a shows this thermally activated behavior for two samples. Furthermore, Figure 14 depicts two different representations of the data for samples on the metallic side of the insulator-to-metal transition referring to the predicted behavior according to Equation 27 (Figure 14b) and Equation 28 (Figure 14c). The predicted universal logarithmic temperature dependence is fulfilled over a large temperature range from room temperature down to about 15 K [54]. Below this temperature deviations occur that could be indicative of the onset of coherent transport as expected for a granular Fermi-liquid (see Figure 14c). However, this latter part needs more thorough investigation at even lower temperatures.

Pt–C structures grown by FEBID provide a particularly valuable example of a nanogranular metal in which the intergrain tunnel coupling strength *g* can be tuned over a wide range so that the insulator-to-metal transition can be approached and passed with excellent control. In [54] a simple graphical analysis was introduced that allows for a quantitative determination of the coupling strength of samples that follow the universal logarithmic dependence on the metallic side. *g*-values between 0.25 and 3 were found with increasing irradiation dose. Several follow-up investigations are at hand to address important aspects for granular metals. The behavior for metallic samples needs to be followed to the sub-Kelvin regime with high data fidelity to check whether the indicated granular-Fermi-liquid behavior is indeed observable. One has to keep in mind that the theoretical model neglects disorder effects which are, of course, present in the samples. It would be desirable to extend these investigations to higher-order transport coefficients, such as the Seebeck effect [55] and also galvanomagnetic quantities (Hall resistance, magnetoresistance) for which also theoretical predictions are available and await experimental verification. With regard to the influence of disorder on the electronic properties of nanogranular metals, studies on artificial granular lattices would be particularly interesting. Initial steps in this regard have been taken in two recent investigations on two-dimensional granular dot-lattices prepared by using the precursor W(CO)_6_ [56–57]. In these experiments a pitch-controlled insulator-to-metal transition was observed. Samples with large pitch (40 nm) clearly showed Arrhenius-like behavior at low-temperatures and indicated the presence of a hard energy gap consistent with the expected Coulomb-blockade energy of the individual nanodots [56]. At this stage it can only be speculated that the dot-size of about 20 nm and the minimum pitch of 20 nm realized in these experiments is too large to allow cotunneling. As a consequence, the expected correlated VRH behavior was not found. Nevertheless, the FEBID technique provides the capability to prepare nanodot lattices in the sub-10 nm regime, which would allow for a thorough comparison of the transport characteristics of disordered and ordered nanogranular metals.

### Nano-granular FEBID sensors

The final section is devoted to the application of FEBID materials for sensor applications, which take particular advantage of the nanogranular microstructure. The applications addressed here are the detection of mechanical strain and magnetic fields employing highly miniaturized FEBID sensor elements.

#### Strain sensing with nanogranular metals

**Physical principles of strain sensing with granular metals**: The concept of strain sensing with granular metals is based on the observation that charge transport is realized via thermally assisted tunnel processes for which the tunneling probability decays exponentially with the intergrain distance. Several techniques for the preparation of granular strain sensors have been established in recent years. The most active areas of research are based on diamond-like carbon (DLC) films with metal inclusions [58] and, since very recently, FEBID-based sensor elements [51]. Although conceptually simple, a theoretical framework with predictive power concerning promising sensor-optimization strategies for this material class has been only recently suggested by one of us [59] and shall be in part very briefly reviewed here.

The strain-dependence of the conductivity or resistivity follows from the derivative

[31]
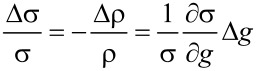


with ρ and σ denoting the (temperature-dependent) resistivity and conductivity, respectively. Employing the expressions for the temperature-dependent conductivity regimes presented in the last section, the respective derivations can be done algebraically. With a view to the largest sensor response, i.e., the largest strain-resistance effect expressed via the gauge factor κ

[32]
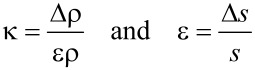


with *s* as the peripheral intergrain spacing, the Arrhenius regime can be identified as the most promising [59]. However, the dependence of the hard energy gap Δ*_M_* (see Equation 29) on the coupling strength *g* is not readily apparent. Theory predicts an exponential functional dependence in the intermediate coupling regime (*gz* ≈ 1, *z*: number of nearest-neighbor grains) as the metallic regime is approached [12]

[33]
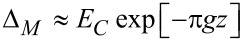


From this dependence and Equation 29 the following derivative can be readily obtained

[34]
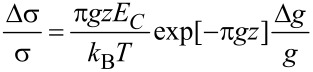


In order to draw a link to the experimentally observed quantity Δρ/ρ(ε) the exponential dependence of the intergrain coupling strength *g* on the intergrain distance *s* has to be explicitly introduced

[35]
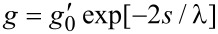


where 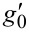 subsumes material-dependent details of the tunnel barrier and λ is the attenuation length of the wave function. The latter coincides with the range for inelastic cotunneling

[36]
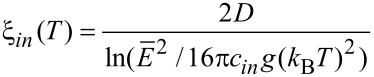


with the boundary condition 1/4 ≤ *c_in_* ≤ 1 and here *c_in_* = 1, if only the short-range part of the Coulomb interaction is important. *D* denotes the average grain diameter. 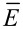 is a measure of the average Coulomb blockade energy of an individual grain and lies within the range *E_C_*/2 ≤ 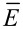 ≤ *E_C_*. The relative change of the coupling strength can now be expressed by the relative change of the grain distance

[37]
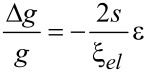


such that the relative conductivity change according to Equation 34 is now fully expressed as a function of the relative length change ε with *s* as a parameter that can be obtained from a suitable relationship between *s* and the metal volume fraction *f*, which depends on the details of the microstructure of the granular metal. In [59] a regular and dense packing of monodisperse spheres (fcc/hcp-like packing) is assumed, which leads to

[38]
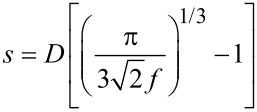


with *z* = 12 nearest neighbors. In this case *f* = 0.7405 for *s* → 0.

With increasing metal content the effective dielectric constant of the granular metal ε*_r_* starts to deviate from that of the insulating matrix. To some degree this can be taken into account by employing an effective-medium theory, such as the Maxwell–Garnett approximation [59]

[39]
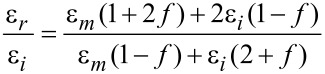


in which ε*_m_* and ε*_i_* denote the dielectric constant of the metal and dielectric matrix, respectively.

**Figure 15:**
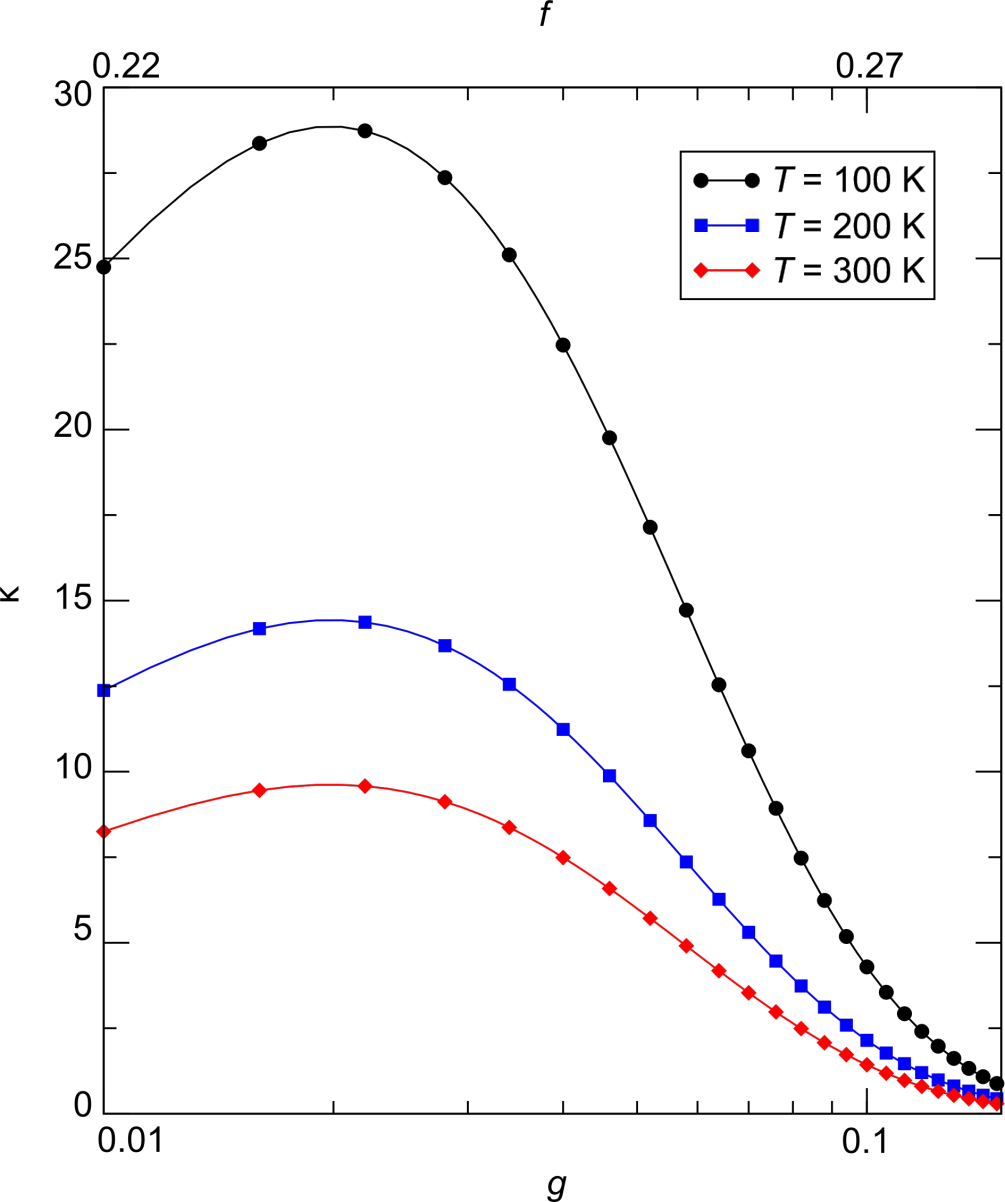
Calculated gauge factor κ as a function of intergrain coupling strength (bottom axis) and metal volume fraction (upper axis) at selected temperatures for the Arrhenius regime at intermediate coupling. Fcc-like packing of Au nanoparticles of 5.5 nm diameter in a dielectric matrix was assumed. The model parameters 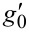 = 3.29, ε*_i_* = 1.02, ε*_m_* = −16400 and 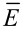 = *E_C_* were used. For details see [59].

In Figure 15 the result of a model calculation in the intermediate coupling Arrhenius regime is shown for three different temperatures. From these calculations a gauge factor of about 10 can be expected at room temperature. This has to be corrected for purely geometric effects caused by the reduction of the sample’s cross section, and its length increase under tensile strain, which leads to an additional strain-resistance effect that adds to the intrinsic gauge factor. The resulting gauge factor then amounts to about 12, which was indeed found in experiments on Pt–C FEBID fabricated strain sensors as is shown in the following subsection. For strain sensors operating in the Arrhenius regime an enhancement of the gauge factor can be expected for smaller grain size and small dielectric constants of the matrix material, as detailed by Huth [59]. Depending on the transport regime, other gauge factors result. In particular, within the metallic regime the intrinsic gauge factor is close to 0 and thus not relevant for applications.

**FEBID-based strain sensors**: Nanogranular strain sensors fabricated by means of FEBID offer a very great potential for miniaturization. Also, they can be realized on many different materials (oxides, polymers, metals with an electrical insulation layer, etc.). In selected areas this is a clear advantage, which is demonstrated here by some recent and unpublished results obtained with regard to the development of microcantilever-based atomic force microscopy for biological applications.

**Figure 16:**
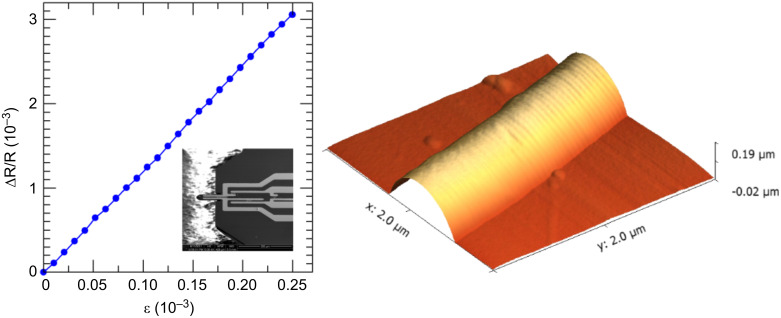
Left: Strain-resistance effect of a Pt–C nanogranular sensor element measured on a test cantilever (see inset). The sensor elements were prepared by FEBID employing the precursor Me_3_Pt(IV)CpMe at 5 keV beam energy, 1.6 nA beam current, 20 nm pitch and 1 μs dwell time. Within a full Wheatstone bridge each individual sensor element had a resistance of 51 kΩ. The Si (100) test cantilever had dimensions of 70 × 35 × 3 μm^3^. Right: Exemplary AFM image of collagen fibril taken in noncontact mode at a resonance frequency of 420 kHz. The images were taken with an adapted MultiMode atomic force microscope with Nanoscope 3a controller at 0.2 Hz line rate.

The strain-resistance effect in FEBID-based sensor elements was shown for the Pt–C system in [51] for the first time. Initial results from the use of sensor elements on cantilevers for dynamic-mode AFM appear in Figure 16. The strain-resistance effect shown in the left part allows for imaging of the surface fine structure of a collagen fibril, which is a proteinaceous fiber and a major component of mammalian connective tissue, such as skin and tendons. As was shown in [51], the voltage noise of the resistive sensor elements follows a 1/*f* frequency dependence and reaches the noise floor at the level of the Johnson noise at about 1 kHz. This frequency response is favorable for dynamic mode AFM applications. In the limit of highly miniaturized cantilevers, to be used in future high-speed AFM approaching line frequencies up to 1000 Hz, the sensor performance can be extrapolated to reach a deflection sensitivity of more than 200 μV/nm at a noise level of about 0.07 nm, for ultrasmall cantilevers with a size of 1 × 0.5 × 0.1 μm^3^. For these cantilever structures, optical readout of the cantilever deflection is not an option anymore.

#### Micro Hall magnetometry with nanogranular metals

**Hall effect in granular metals**: The detection of spatially inhomogeneous magnetic fields, such as the dipolar stray fields obtained from magnetic beads for biological and medical applications or in magnetic media, relies on the availability of nanometer-sized magnetic sensor elements. Nanogranular metals with ferromagnetic grains can provide excellent detection sensitivity due to their large interfacial area per unit volume, which leads to a strong increase of the surface scattering rate and results in a strongly enhanced extraordinary Hall effect (EHE) as the insulator-to-metal transition is approached from the insulating side. More specifically, the Hall resistivity ρ*_H_* in a ferromagnetic metal has contributions that stem from the Lorentz force acting on the charge carriers, i.e., the ordinary Hall effect (OHE), and the EHE, which is proportional to the spontaneous magnetization [60]

[40]
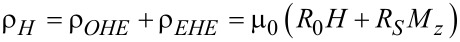


where *H* denotes the applied magnetic field aligned perpendicularly to the Hall device, *M_z_* is the spontaneous magnetization in the field direction 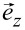, *R*_0_ and *R_S_* are the ordinary and spontaneous Hall constants, respectively, and μ_0_ is the magnetic permeability of the vacuum. In metals the OHE is negligible compared to the EHE, so that the saturation field of the magnetic response defines the upper limit of the working range of such a Hall device. The device yields a signal proportional to the local magnetization *M*(*H*; *x*, *y*)*_z_* averaged over the cross section of the nanostructured Hall sensing area. The sensitive dependence of the EHE on surface scattering is due to the relation between *ρ_EHE_* and the longitudinal resistivity ρ with contributions from skew and side-jump scattering [60]

[41]
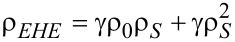


Here, ρ has been decomposed by using Matthiessen’s rule in the spin-independent part ρ_0_ and the magnetic part ρ*_S_*.

**FEBID-based Hall sensors**: Submicrometer Hall devices prepared by FEBID employing the precursor Co_2_(CO)_8_ were first described by Boero and collaborators [38]. This work was later extended towards optimization of the Hall sensitivity by Gabureac et al. [39]. The devices in standard Hall-cross geometry had a thickness between a few tens up to a few hundreds of nanometers and widths between 200 and 500 nm. It was found that the room temperature *M_z_*(*H*) curves could be excellently described by a Langevin fit, indicating superparamagnetic behavior

[42]
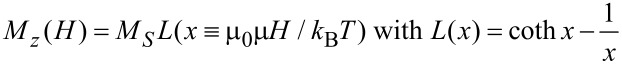


μ denotes the averaged magnetic moment, which can be deduced by fitting the data according to Equation 40 with *M_z_* given by Equation 42. In the linear region of the *M_z_*(*H*) characteristics the supply-current-related field sensitivity *S_I_* = *I*^−1^*dV_H_*/*d*(μ_0_*H*), with *I* denoting the bias current and *V_H_* the Hall voltage, was found to be 0.15 Ω/T at 10 mA current for samples with about 65% Co content. This translated to a field-detection limit of 3 μT/Hz^1/2^. The frequency-dependent voltage noise of the Hall device was found to follow a 1/*f* behavior hitting the thermal noise limit in the 100 kHz range for the largest bias currents.

The observed field detection limit of the Co–C Hall sensors is by a factor of about 100 worse than those which can be obtained with state-of-the-art InAsSb quantum-well structures [61]. However, the relevant quantity is the minimum detectable magnetic flux Φ*_min_* = *B_min_A* (*A*: sensing area) when considering the demands on a micro-Hall sensor, which is typically exposed to a highly inhomogeneous magnetic field distribution. It was found that for the Co–C Hall devices with the smallest width of about 100 nm this amounted to 4.5 × 10^−6^ Φ_0_, with Φ_0_ = *h*/2*e* the magnetic flux quantum, under optimal conditions which is about one order of magnitude better than what can be realized with semiconductor-quantum-well structures [39].

## Conclusion

In this review a selected summary of recent developments in the use of FEBID-structures in basic and applied research has been presented. The addressed topics were fundamental questions relating to the nature of the charge transport in nanogranular metals close to the insulator-to-metal transition, the extension of FEBID to a multiprecursor technique for the direct nanostructure formation of granular alloys and intermetallic compounds, and finally to sensor applications, which benefit from this same granular structure. The authors consider these new developments as very promising for the development of the FEBID technique towards the fabrication of functional nanostructures, albeit the aspect of long-time stability of the transport properties certainly needs more attention [62]. On the other hand, when considering the development over the past two decades it can be stated that the holy grail of FEBID has been the identification of deposition protocols to obtain the purest metallic nanostructures possible. Presently, this has been achieved for a very limited group of precursors, e.g., Co_2_(CO)_8_ or Fe(CO)_5_ (under UHV conditions) and not without problems, such as precursor instability or autocatalytic growth contributions, which limit the ultimately achievable resolution [63]. Nevertheless, the availability of FEBID processes for pure metallic structures would without any doubt render this technique the most versatile direct nanostructure fabrication technique in many fields of nanotechnology, be it in basic or applied research. Although this is certainly a valid argument, from a broader perspective FEBID holds the potential to become the basic technology of an electron-beam-induced and -controlled chemistry on the nanometer scale. The aspect of control is the critical issue in this regard. Very little research has been carried out concerning the details of the dissociation pathways for FEBID-relevant precursors, be it experimentally or theoretically [17,64]. This certainly needs to be intensified to provide the basis for the next step of controlling the dissociation process under electron impact, e.g., by providing supporting chemical agents that saturate free bonds of organic dissociation products, thus preventing their polymerization and keeping them sufficiently volatile to be eventually pumped away. There appears not to be a principle limitation in developing a specialized surface chemistry that is triggered by electrons but nevertheless can be controlled to a significant degree by supplying a suitable chemical environment aiming for an optimized product yield, e.g., a pure simple metal or alloy. From this perspective FEBID will have to move towards a better microscopic understanding of all relevant processes in a controlled environment, i.e., under UHV conditions and augmented by a selection of surface science analysis techniques. One may hope that results from research under these much better controlled conditions (see e.g., [65–66]) will also be helpful to optimize FEBID processes in the standard SEM environment where it is already today a most attractive technique for structure formation on the nanometer scale.
